# Supplementary material for: CasNano: An ultracompact CRISPR-Cas12f with a GC-rich PAM for epigenome editing with therapeutic potential
Source: Mol Ther Nucleic Acids. 2026 Sep 18;37(4):103106. doi: 10.1016/j.omtn.2026.103106 (PMC13670948; doi:10.1016/j.omtn.2026.103106)
Supplement: Supplementary file 1 [file mmc1.pdf]

OMTN, Volume *37*

Supplemental information

CasNano: An ultracompact CRISPR-Cas12f with a GC-rich PAM for epigenome editing with therapeutic potential

Dongkyeong Kim, Chia-Wei Huang, Richard Pan, Junming Qian, Xuebing Wu, Orrin Devinsky, Yi-Ting Tsai, and Shawn Liu

Table S1. The 22 candidate residues evaluated in the initial activity screen. The 10 residues with >80% wild-type activity (advanced to iterative engineering) are underlined.

Order	Amino acid
1	Y48
2	A50
3	T66
4	S98
5	T99
6	E109
7	N114
8	F119
9	S131
10	G176
11	E181
12	D183
13	K184
14	N195
15	K220
16	A223
17	G266
18	A276
19	Y306
20	T307
21	K318
22	D353

Table S2. CasNano Sequences

Name	Description	Protein sequence	Length
WT BlCas12f	The original, unmodified wild-type Cas12f protein sequence derived from *Blautia lenta*.	MITVRKVKLIVNSEEAEEINRTYKFIRDSMYAQYQGLNRCMGYLLSGYYANGMDIKSDGFKNHMKTIKNSLNIFDDINFGIGIDSKSAITQKVKKDFSTSLKNGLAKGERGATNYKRNFPLMTRGRDVKISYLEDTNTFVIKWVNKIEFKVILGQKDNIELSHTLHKIINKEYTLGQCTFEFDKNNKLLLALNINIPDNLISKNKEIIPGRVLGVDLGVKVPAMICLNDNTFIKKSIGSYNEFFKVRSQFKARRERLYKQLESSNGGKGRKHKLKATMQFRDKEKNFARTYNHFLSKNIIEFAQKYTCETINLEELNKKGFDNNLLGKWGYYQLQSMIEYKAERVGIKVKYVDPAFTSQTCSKCGYVDEENRITQDKFECQKCGFTLNADHNAAINIARK	400
CasNano V11	The final engineered CasNano variant incorporating all optimized mutations for enhanced targeting efficiency.	MITVRKVKLRVNSEEAEEINRTYKFIRDSMYAQYQGLNRCMGYLLSGYYANGMDIKSDGFKNHMKTIKNSLNIFDKINFGIGIDSKSAITQKVKKDFSTSLKNGLAKGERNATNYKRNFPLMTRGRDVKICYLEDTNTFVIKWVNKIEFKVILGQKDNRELSHTLHKIINKEYTLGQCTFQFTKNNKLLLALNIKVPDNLISKNKEIIPGRVLGVDLGVKVPAMICLNDNTFIKKSIGSYNEFFKVRSQFKARRERLYKQLESSNGGKGRKHKLKATMQFRDKEKNFARTYNHFLSKNIIEFAQKKKCETINLEELNKKGFDNNLLGKWGYYQLQSMIEYKAERVGIKVKYVDPAFTSQTCSKCGYVDEENRITQDKFECQKCGFTLNADHNAAINIARK	400

dCasNano V11	The catalytically dead version of CasNano V11 (harboring the D216A and D390A mutations). This variant lacks nuclease cleavage activity but retains full DNA-binding capability, serving as the versatile platform for fusion with transcriptional and epigenetic effectors (e.g., VPR, DNMT3A, TET1).	MITVRKVKLRVNSEEAEEINRTYKFIRDSMYAQYQGLNRCMGYLLSGYYANGMDIKSDGFKNHMKTIKNSLNIFDKINFGIGIDSKSAITQKVKKDFSTSLKNGLAKGERNATNYKRNFPLMTRGRDVKICYLEDTNTFVIKWVNKIEFKVILGQKDNRELSHTLHKIINKEYTLGQCTFQFTKNNKLLLALNIKVPDNLISKNKEIIPGRVLGVALGVKVPAMICLNDNTFIKKSIGSYNEFFKVRSQFKARRERLYKQLESSNGGKGRKHKLKATMQFRDKEKNFARTYNHFLSKNIIEFAQKKKCETINLEELNKKGFDNNLLGKWGYYQLQSMIEYKAERVGIKVKYVDPAFTSQTCSKCGYVDEENRITQDKFECQKCGFTLNAAHNAAINIARK	400

Table S3. gRNA Scaffold

Variant	Scaffold sequence	Length
WT full length	CAGGATAAATTTGAATGCCAAAAGTGTGGCTTTACTCTTAA TGCAGATCATAATGCAGCAATAAATATTGCACGTAAGTAAA CATTGATAAAACTAACCTTTATTTACAAATTTAAAATAATGC AATATAATTGTATTGATGTTATATATAAATATATAGCAGTTA CGGTAACTTAAAGTGCCGAAGGCTGAGGAGATGGATTAAA TATATAAGGTTTTGACCAACTATATACCATCATCACTCGGT AAGGGTTAATCCTAACAATGTGTGACCGTTAGGCGTTCCA AAATTGAATAATTATATTAGTTTATTGTCTATCATAGATAGT GTAAAAAACACAGGGGGTAGACAAAATAAGTAATATAAGT ATATGGTAGCTA	381
Variant 1	TAATGCAATATAATTGTATTGATGTTATATATAAATATATAG CAGTTACGGTAACTTAAAGTGCCGAAGGCTGAGGAGATG GATTAAATATATAAGGTTTTGACCAACTATATACCATCATCA CTCGGTAAGGGTTAATCCTAACAATGTGTGACCGTTAGGC GTTCCAAAGAAATGGAATGTTAAT	187
Variant 3	GACGGTAACTTAAAGTGCCGAAGGCTGAGGAGATGGATTA AATATATAAGGGTTTGCCCAACTATATACCATCATCACTCG GTAAGGGTTAATCCTAACAATGTGTGACCGTTAGGCGTTC CAAAGAAATGGAACGTTAAT	141
Variant 5	GACGGTAACTTAAAGTGCCGAAGGCTGAGGAGATGGATTA AATATATAAGGGTTTGCCCAACTATATACCATCATCACTCG GTAAGGGTTAATCCTAACAATGTGTGACCGTTAGGCGTTC CAAAGAAATGGAATGTTAAT	141
Variant 14	GACGGTAACTTAAAGTGCCGAAGGCTGAGGAGATGGATTA AATATATAAGGTTTTGACCAACTATATACCATCATCACTCG GTAAGGGTTAATCCTAACAATGTGTGACCGTTAGGCGTTC CAAAGAAATGGAACGTTAAT	141
Variant 18	GCGTAACTTAAAGTGCGAAGGCTGAGGAGATGGATTAAAT ATATAAGGTTTCGACCAACTATATACCATCATCACTCGGTA AGGGTTAATCCTAACAATGTGTGACCGTTAGGCGTTCCGA AAGGAACGTTAAT	134

Table S4. gRNAs

	Target	gRNA spacer sequence	Lenth
1	Scramble	GATATGCTTCTAGATGAGAA	20
2	TetO(sense)	CTCCCTATCAGTGATAGAGA	20
3	TetO(antisense)	TCTCTATCACTGATAGGGAG	20
4	HBG1(sense)	GGCTAAACTCCACCCATGGGT	21
5	HBG1(sense)	ATAGTCTTAGAGTATCCAGTG	21
6	HBG1(sense)	GTGAGGCCAGGGGCCGGCGGC	21
7	HBG1(sense)	TGGGTCATTTCACAGAGG	18
8	HBG1(antisense)	ACCCATGGGTGGAGTTTAGCC	21
9	HBG1(antisense)	CACTGGATACTCTAAGACTAT	21
10	HBG1(antisense)	GCCGCCGGCCCCTGGCCTCAC	21
11	HBG1(antisense)	CCTCTGTGAAATGACCCA	18
12	IL1RN(sense)	TGTACTCTCTGAGGTGCTC	19
13	IL1RN(sense)	CCTGGAAACTGGGCCTGCT	19
14	IL1RN(sense)	GAGTCACCCTCCTGGAAACT	20
15	IL1RN(sense)	CTGCCTTGCTGCAGTCACAGAA	22
16	IL1RN(antisense)	GAGCACCTCAGAGAGTACA	19
17	IL1RN(antisense)	AGCAGGCCCAGTTTCCAGG	19
18	IL1RN(antisense)	AGTTTCCAGGAGGGTGACTC	20
19	IL1RN(antisense)	TTCTGTGACTGCAGCAAGGCAG	22
20	GAPDH-mSnrpn	TTGCCTGGGACGCATGCG	18
21	GAPDH-mSnrpn	TTGCGGCAAGACTAGCGC	18
22	GAPDH-mSnrpn	GCTGACCTTCCTCGCTCC	18
23	DAZL-Snrpn	TTGCCTGGGACGCATGCG	18
24	DAZL-Snrpn	TTGCGGCAAGACTAGCGC	18
25	DAZL-Snrpn	GCTGACCTTCCTCGCTCC	18
26	MeCP2	CAGCCCTCTCTCCGAGAGG	19
27	MeCP2	CTCACAGTCTCTCCTCCTCG	20
28	MeCP2	CTAAACCAGTCCCTCCGCGC	20

Table S5. Primers

Primers	Sequence	Length
IL1RN-F	GGAATCCATGGAGGGAAGAT	20
IL1RN-R	TGTTCTCGCTCAGGTCAGTG	20
HBG1-F	GTCCTGGTCACCAGAGCCT	19
HBG1-R	ACCACTGCTAACTGAAAGAGAC	22
GAPDH-Snrpn-R	TTAGGTTAGTTGAGAGAATTTTAGAATA	28
GAPDH-Snrpn-F	CTCCTTAATAATAACCATATTATCCTCC	28
DAZL-Snrpn-R	GGGAGGAGTAGAGAGTATTAGGG	23
DAZL-Snrpn-F	CTCCTCGCCCTTACTCACCATAATAAC	27

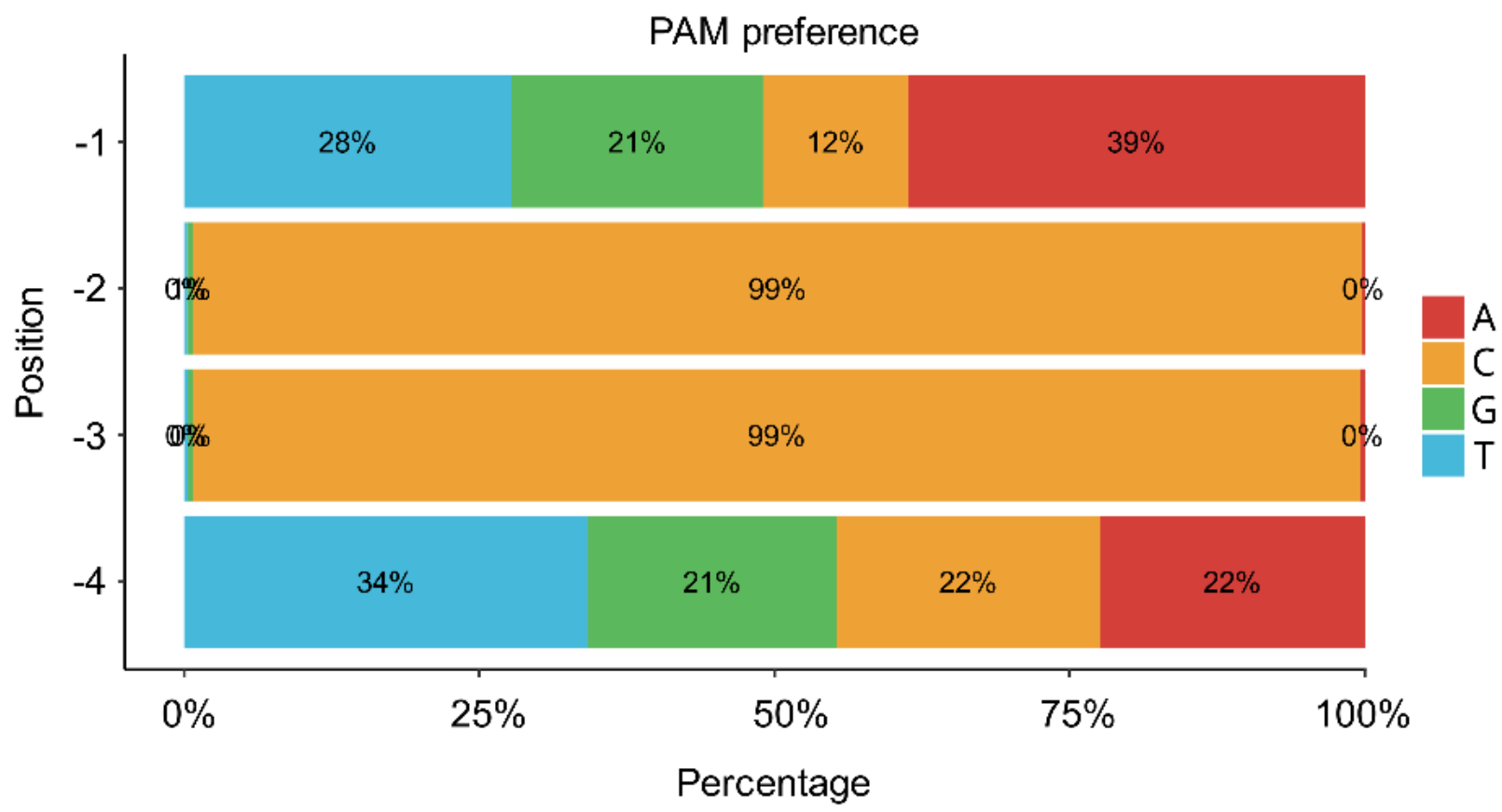

Figure S1. Characterization of CasNano PAM Preference.

Bar chart showing the nucleotide preference at the -1 to -4 positions of the protospacer adjacent motif (PAM). A library of reporter plasmids, each containing a unique 4-bp 5'-NNNN-3' sequence upstream of the TetO target site, was co-transfected into HEK293T cells with dCasNano-VPR and a TetO-targeting sgRNA. RFP intensity was measured by flow cytometry, and the relative activity for each nucleotide at each position was calculated to determine the preference. The results show a strict requirement for Cytosine (C) at positions -2 and -3.

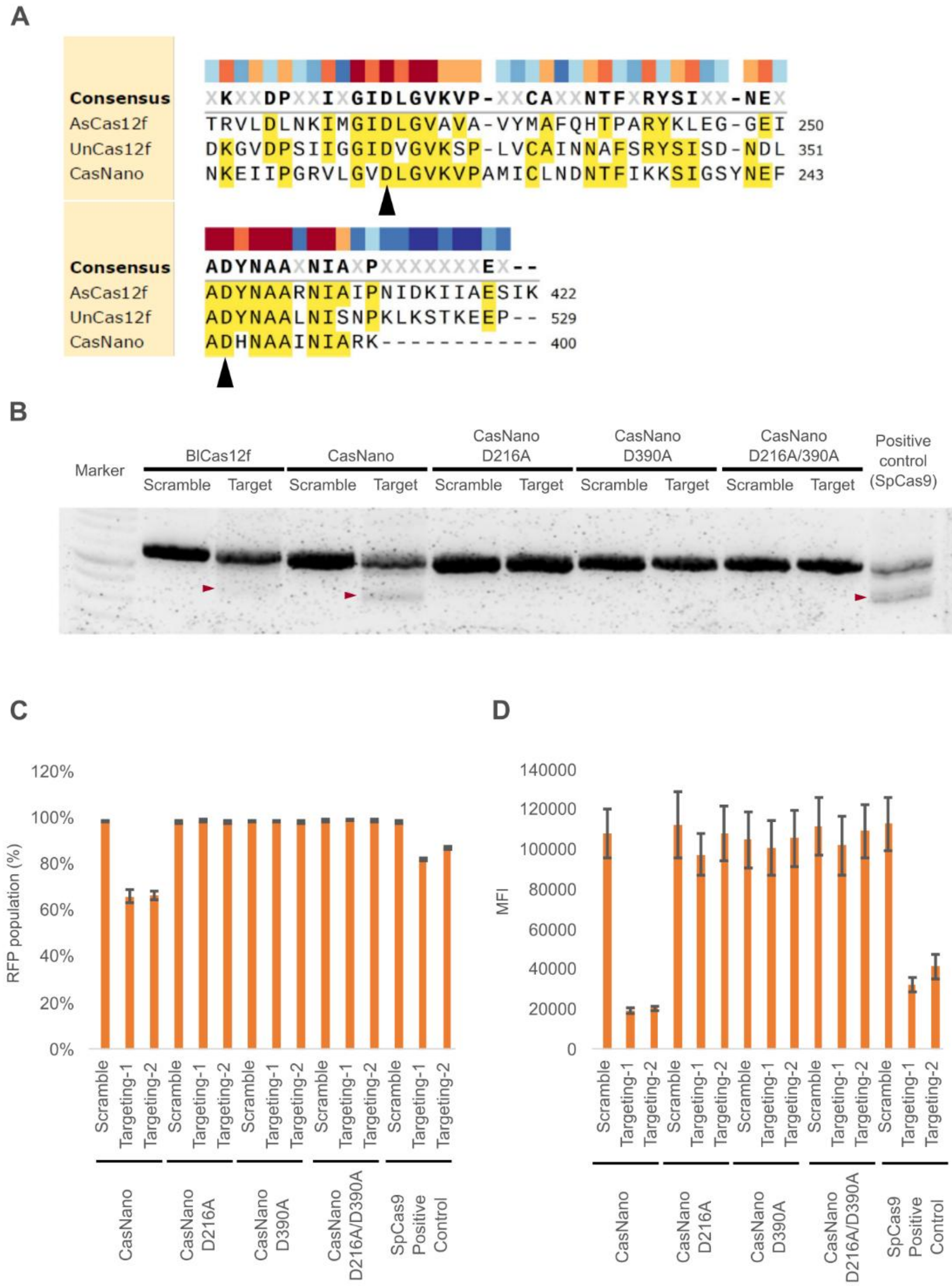
A
Consensus XKXXDPXXIXGIDLGVKVP-XXCAXXNTFXRYSIXX-NEX
AsCas12f TRVLDLNKIMGIDLGVAVA-VYMAFQHTPARYKLEG-GEI 250
UnCas12f DKGVDPSIIGGIDVGVKSP-LVCAINNAFSRYSISD-NDL 351
CasNano NKEIIPGRVLGVDLGVKVPAMICLNDNTFIKKSIGSYNEF 243
Consensus ADYNAAXNIAXPXXXXXXXEX--
AsCas12f ADYNAARNIAIPNIDKIIAESIK 422
UnCas12f ADYNAALNISNPKLKSTKEEP-- 529
CasNano ADHNAAINIARK----------- 400
B
Marker
BlCas12f
CasNano
CasNano D216A
CasNano D390A
CasNano D216A/390A
Scramble
Target
Positive control (SpCas9)
C
RFP population (%)
120%
100%
80%
60%
40%
20%
0%
Scramble
Targeting-1
Targeting-2
CasNano
CasNano D216A
CasNano D390A
CasNano D216A/D390A
SpCas9 Positive Control
D
MFI
140000
120000
100000
80000
60000
40000
20000
0
Scramble
Targeting-1
Targeting-2
CasNano
CasNano D216A
CasNano D390A
CasNano D216A/D390A
SpCas9 Positive Control

Figure S2. Determination of Catalytic Amino Acids of CasNano Nuclease Activity.

(A) Protein sequence alignment of the RuvC domain from CasNano with AsCas12f and UnCas12f, highlighting the predicted catalytic aspartate residues (D216 and D390, indicated).

(B) Surveyor nuclease assay showing indel formation. Wild-type BlCas12f and the engineered CasNano (V11) were transfected into HEK293 cells with either a scramble control sgRNA or two different sgRNAs targeting the RFP gene. Cleaved DNA fragments indicative of NHEJ are marked with an arrow. Semi-quantitative densitometric analysis using ImageJ confirmed that the indel frequency of wild-type BlCas12f was near background levels (~0.10%). The engineered CasNano variant exhibited a weak nuclease activity (~7.45%), which was completely abolished (0%) by the D216A and/or D390A mutations. SpCas9 was used as a positive control (~28.90%).

(C and D) Quantification of nuclease activity via a functional knockout assay in TetO-RFP reporter cells. Bar charts show the percentage of RFP-positive cells (C) and the mean fluorescence intensity (MFI) of RFP (D) after transfection with nuclease-active CasNano or variants with single (D216A, D390A) or double (D216A/D390A) catalytic mutations. A decrease in RFP signal indicates successful cleavage and NHEJ-mediated gene disruption. Nuclease-active SpCas9 serves as a positive control.

All data are represented as mean ± SD for n = 3 biological replicates.

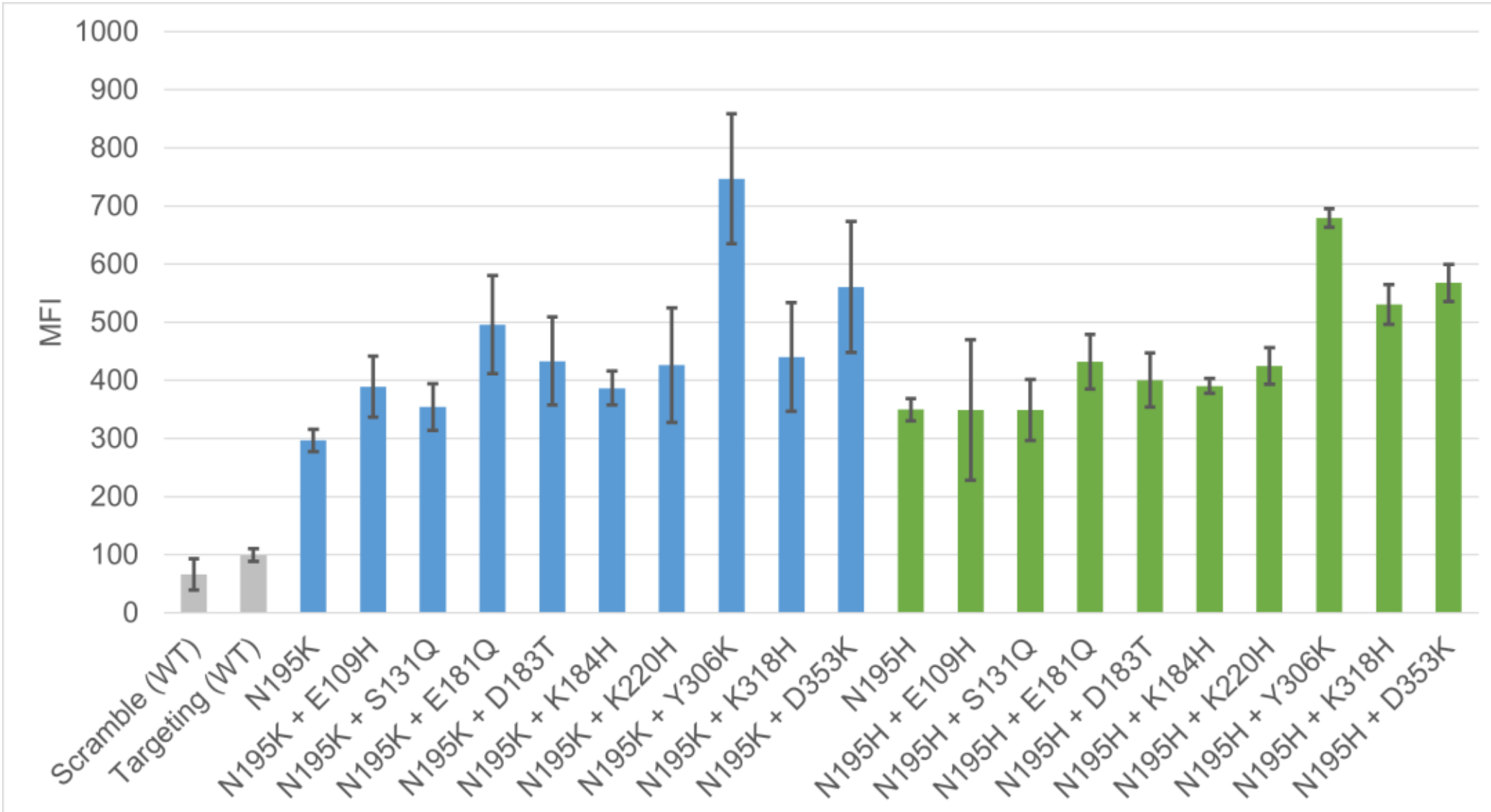

Figure S3. Combinatorial pairwise testing of N195K vs N195H as the V1 scaffold of dBlCas12f-VPR.

Reporter activation of the indicated dBlCas12f-VPR variants on the TRE3G-RFP reporter in HEK293T cells, measured by flow cytometry 72 h post-transfection. Bars show the mean fluorescence intensity (MFI) of RFP. Gray: wild-type dBlCas12f-VPR with either a scramble or a targeting sgRNA, providing the scramble background and the unengineered baseline-activity controls. Blue: the N195K-base series, comprising the single mutation N195K and N195K paired in turn with each of the nine top single-mutation candidates retained from saturated mutagenesis (E109H, S131Q, E181Q, D183T, K184H, K220H, Y306K, K318H, D353K; Figure 1B). Green: the corresponding N195H-base series, with the same nine pairings on the alternative N195H base. Data are mean ± SD for n = 2 biological replicates.

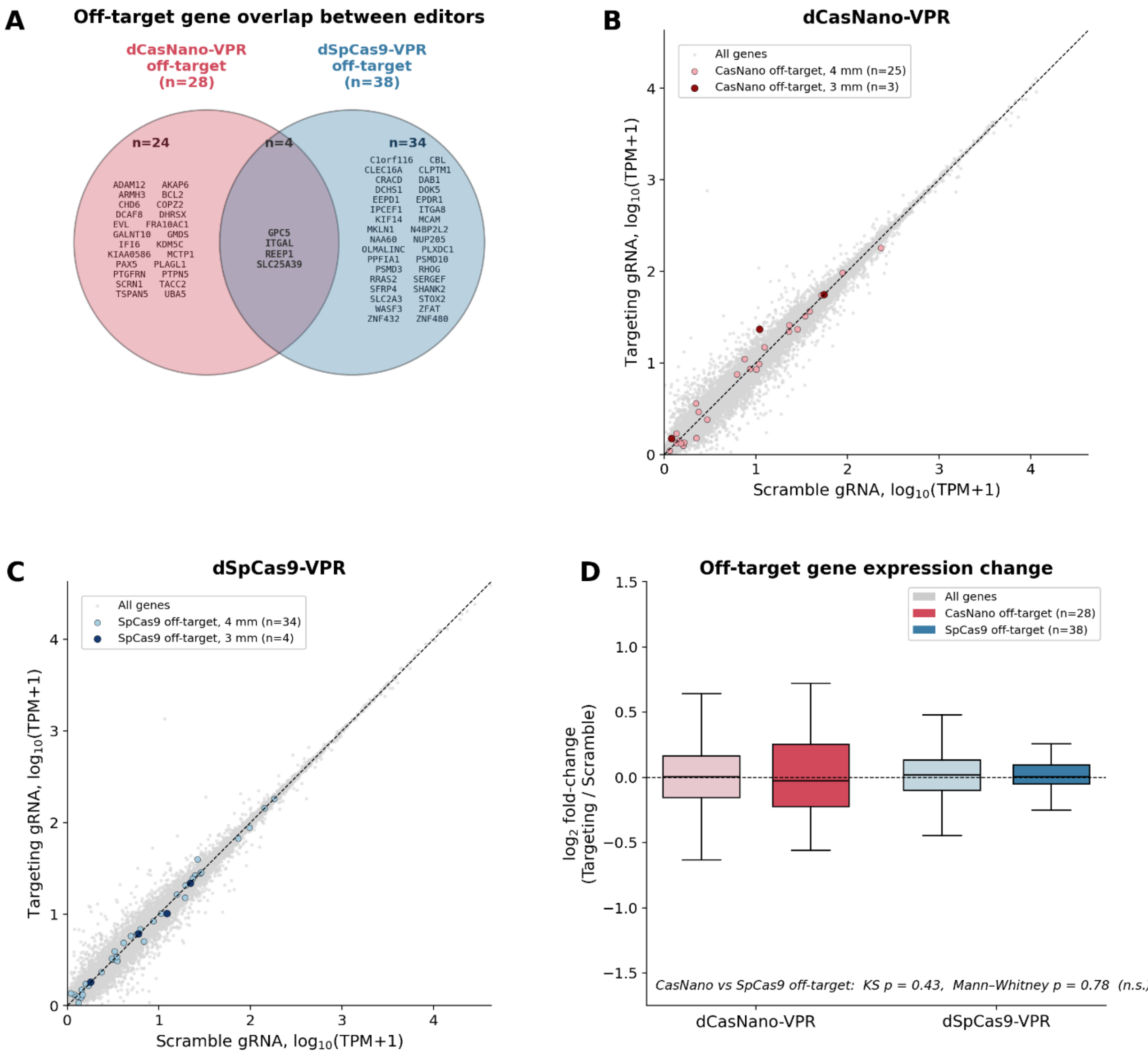

Figure S4. Genome-wide transcriptional specificity of dCasNano-VPR and dSpCas9-VPR at predicted off-target loci.

(A) Overlap between the predicted off-target gene sets of the two editors. Candidate sites within four mismatches of the shared spacer were identified separately for each system, reflecting their distinct PAM requirements. (B and C) Gene expression (log10(TPM+1)) under Targeting versus Scramble gRNA conditions for dCasNano-VPR (B) and dSpCas9-VPR (C). Each editor's own predicted off-target genes are highlighted by mismatch number; the remaining transcriptome is shown in gray. (D) Box plots of log2 fold-change (Targeting/Scramble) for all genes and for each editor's predicted off-target genes. Kolmogorov–Smirnov and Mann–Whitney p values compare the two off-target gene sets.

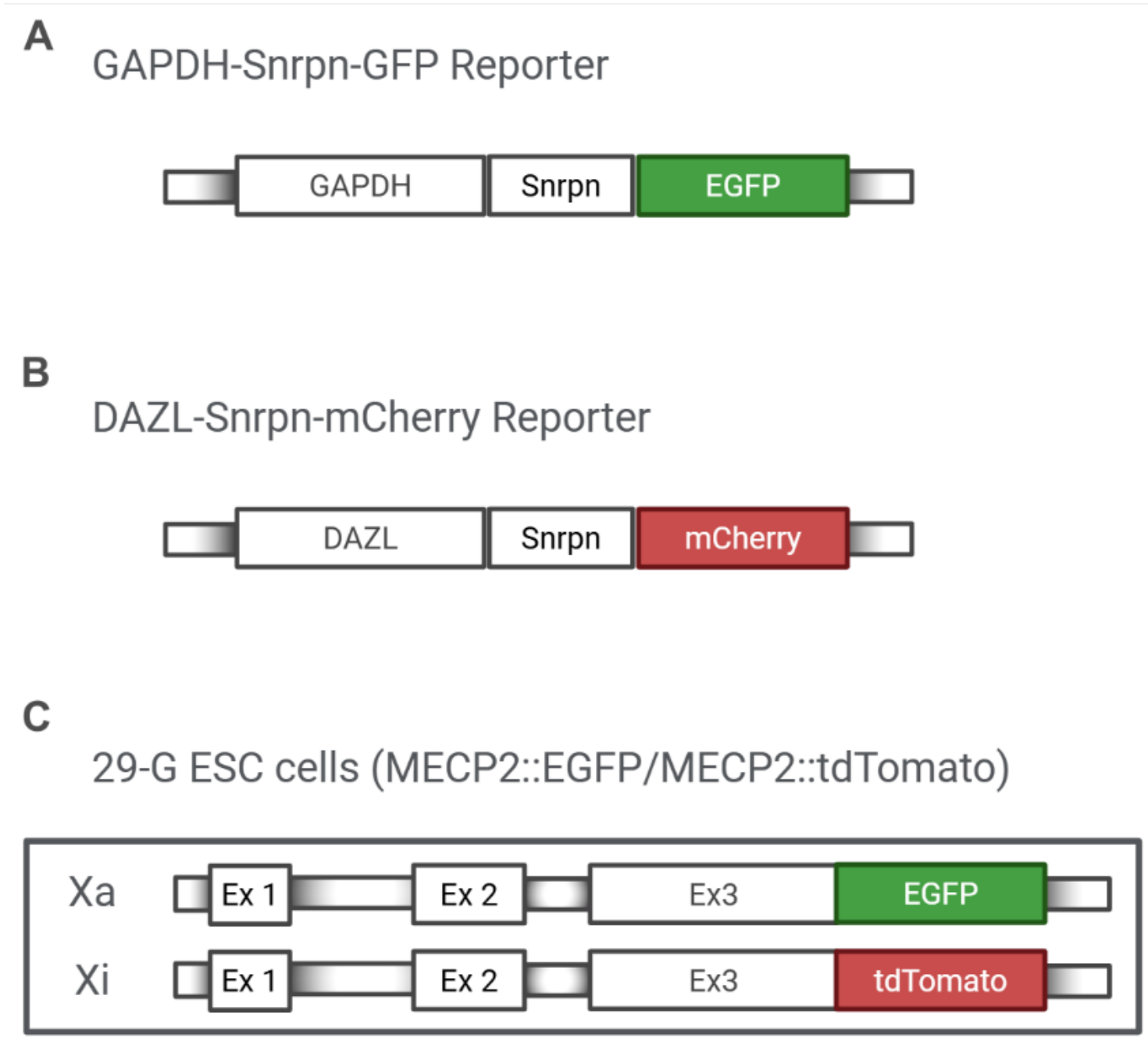

Figure S5. Schematics of Reporter Constructs Used for Epigenetic Editing.

(A) Diagram of the GAPDH-Snrpn-EGFP reporter construct integrated into the HEK293 genome. The constitutively active GAPDH promoter keeps the adjacent Snrpn promoter from silencing and maintain the expression of a downstream EGFP reporter.

(B) Diagram of the DAZL-Snrpn-mCherry reporter construct. The heavily methylated DAZL promoter leads to silencing of the linked Snrpn promoter and the mCherry reporter.

(C) Diagram of the 29-G hESC reporter line for the *MECP2* locus. An EGFP reporter is fused in-frame to the end of exon 3 on the active X chromosome (Xa), while a tdTomato reporter is fused to the end of exon 3 on the inactive X chromosome (Xi), which is silenced by DNA methylation.

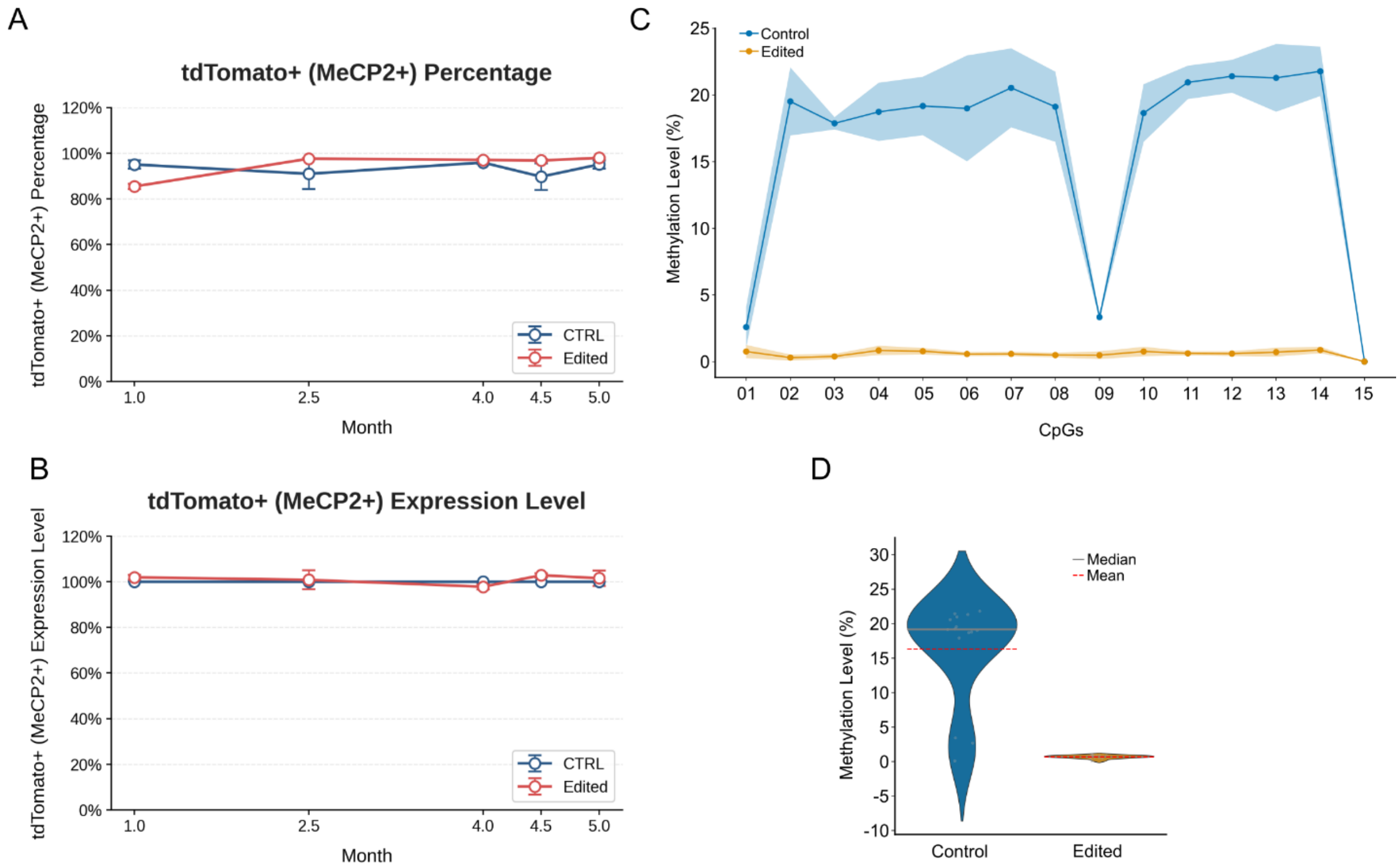

Figure S6. Long-term durability of CasNano-mediated targeted demethylation.

(A and B) Time-course flow cytometry analysis of 29-G hESCs edited with TET1-dCasNano, monitored over a 5-month period. (A) Percentage of tdTomato (MECP2)-positive cells in the culture. (B) Relative tdTomato (MECP2) expression level (MFI). The control for (A) and (B) is the 29-R hESC line, in which the MECP2-tdTomato allele is constitutively expressed; it defines full reactivation, and values in (B) are expressed relative to this line (29-R hESC). (C and D) Bisulfite sequencing analysis of MECP2 promoter CpG methylation at the 5-month time point in TET1-dCasNano-edited versus unedited 29-G hESCs. The control for (C) and (D) is unedited 29-G hESCs, which retain a methylated MECP2 promoter and a silenced tdTomato allele. (C) Per-CpG methylation level (%) across 15 anchor CpG positions spanning the targeted MECP2 promoter region; lines indicate group means, shaded ribbons indicate SD across biological replicates. (D)

Violin plot of methylation level (%) across all profiled CpG positions, with median and mean indicated. (N=5)

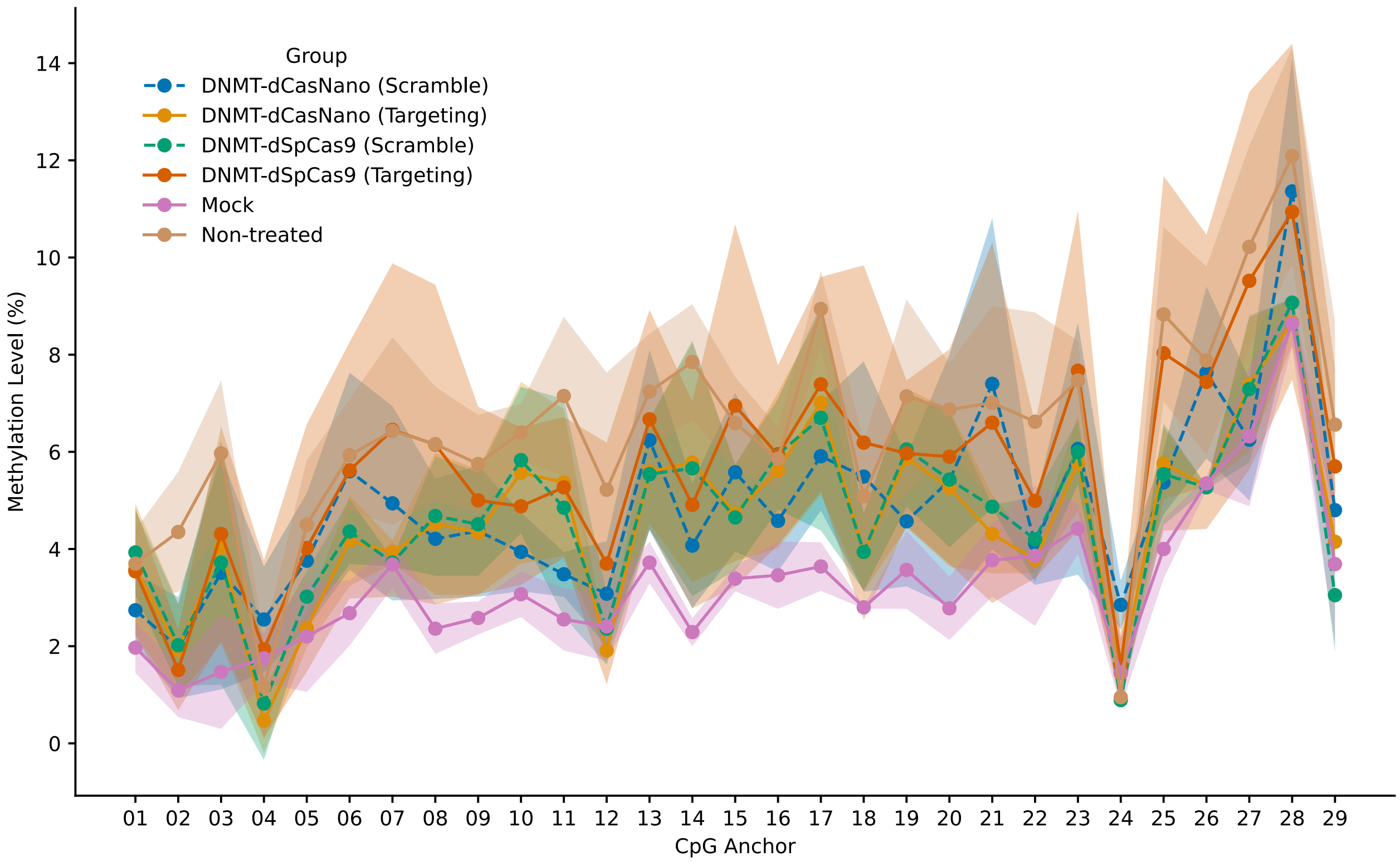

Figure S7. Durability assessment of DNMT3A-dCasNano-mediated methylation in HEK293T cells.

Methylation level (%) at 29 anchor CpG positions across the Snrpn promoter region in the GAPDH-Snrpn-EGFP HEK293T reporter line at 2 weeks post-transient transfection. Lines indicate group means; shaded ribbons indicate SD across biological replicates.
