# Supplementary material for: CasNano: An ultracompact CRISPR-Cas12f with a GC-rich PAM for epigenome editing with therapeutic potential
Source: Mol Ther Nucleic Acids. 2026 Sep 18;37(4):103106. doi: 10.1016/j.omtn.2026.103106 (PMC13670948; doi:10.1016/j.omtn.2026.103106)
Supplement: Supplementary file 2 [file mmc2.pdf]

Molecular Therapy
Nucleic Acids
Original Article

CasNano: An ultracompact CRISPR-Cas12f with a GC-rich PAM for epigenome editing with therapeutic potential

Dongkyeong Kim,[1,4] Chia-Wei Huang,[1,4] Richard Pan,[3] Junming Qian,[3] Xuebing Wu,[2] Orrin Devinsky,[1] Yi-Ting Tsai,[1] and Shawn Liu[3]

[1]Epitor Therapeutics, Inc., New York, NY 10032, USA; [2]Department of Medicine and Department of Systems Biology, New York, NY 10032, USA; [3]Department of Physiology and Cellular Biophysics, Columbia University, New York, NY 10032, USA

Clinical use of CRISPR-Cas proteins for genome engineering is constrained by their large size, limiting *in vivo* delivery. To address this, we engineered an ultracompact and versatile platform from a minimally active, 400-amino acid Cas12f protein from *Blautia lenta*. A multi-stage strategy of structure-guided protein mutagenesis and systematic single guide RNA (sgRNA) optimization transformed BlCas12f into a "CasNano" as a potent genome targeting tool for mammalian cells. When a catalytically dead CasNano (dCasNano) is fused to a VPR domain, it activates endogenous genes with an efficiency and specificity comparable or superior to the benchmark SpCas9 system for the guide RNAs (gRNAs) and loci tested under equal-mass plasmid transfection; as editor expression was not quantified and stoichiometric differences would, in any case, preclude equivalent normalization, these comparisons describe functional output rather than intrinsic activity. We established the platform's versatility for bidirectional epigenetic editing by fusing dCasNano to a DNA methyltransferase (DNMT3A) for gene silencing or a dioxygenase (TET1) for gene activation. TET1-mediated reactivation persisted for 5 months in human embryonic stem cells (hESCs), whereas DNMT3A-deposited methylation was not retained at 2 weeks in a dividing reporter line. CasNano's small size and distinct CCN PAM sequence highlight its potential for future therapeutic applications constrained by viral delivery cargo capacity.

INTRODUCTION

CRISPR-Cas technologies have transformed genome modifications in living cells, advancing basic and clinical science. *Streptococcus pyogenes* Cas9 (SpCas9)-based systems enable precise genome edits, while their nuclease-deactivated counterparts (dCas), fused to effector domains, can modify gene expression via targeted transcriptional and epigenetic modifications.[1] Translating nuclease systems for clinical use has been limited to *ex vivo* editing (e.g., blood disorders) and circumscribed targets with easy access (e.g., eye) since their large size limits packaging into viral capsids and biodistribution in large targets such as the brain.[2–4] Delivery vectors such as the adeno-associated virus (AAV) can only carry ∼4.7 kb, often exceeded by the Cas protein coding sequence, particularly when fused to functional effectors.[5] Smaller molecular size is also advantageous for non-viral delivery methods such as lipid nanoparticles (LNPs), where a more compact mRNA improves packaging efficiency and manufacturing consistency.[6,7] Smaller LNPs may also improve biodistribution.[8–10] These challenges underscore the need for compact and efficient CRISPR-based systems to expand the therapeutic potential of genome engineering.

Commonly used effectors such as SpCas9 (∼1,368 aa) and Cas12a/Cpf1 (∼1,300 aa) are particularly large.[11,12] Even smaller orthologs that have been adapted for use, such as SaCas9 (∼1,053 aa), still occupy a significant portion of the AAV payload capacity. This has driven the exploration of smaller systems, such as Cas12e or the ultracompact Cas12f family, with Cas proteins such as CasX (∼980 aa), UnCas12f1 (CasMINI, ∼529 aa), and the recently engineered AsCas12f1 variants (HKRA/YHAM, ∼422 aa).[13–16] However, these miniature proteins require long AT-rich PAM sequences (e.g., SaCas9: 5′-NNGRRT-3′, CasX: 5'-TTCN-3' PAM, and CasMINI: 5'-TTTR-3' PAM), which limits both the number of targetable genomic sites and therapeutic modification of gene expression in GC-rich promoters and enhancers.[13–15]

Here, we describe the engineering of CasNano, a novel miniature Cas protein from the Cas12f family that is compact in size, efficient in targeting, and has a favorable CCN PAM sequence. Using the minimally active BlCas12f as our base, we created a potent and specific platform to engineer the mammalian genome. When our dCasNano is fused to a transcriptional activator, it drives exceptionally high

Received 10 November 2025; accepted 16 September 2026;
https://doi.org/10.1016/j.omtn.2026.103106.

[4]These authors contributed equally

Correspondence: Yi-Ting Tsai, Epitor Therapeutics, Inc.
E-mail: woofung@gmail.com
Correspondence: Shawn Liu, Department of Physiology and Cellular Biophysics, Columbia University.
E-mail: sl4738@cumc.columbia.edu

Published by Elsevier Inc. on behalf of The American Society of Gene and Cell Therapy.
This is an open access article under the CC BY license (http://creativecommons.org/licenses/by/4.0/).

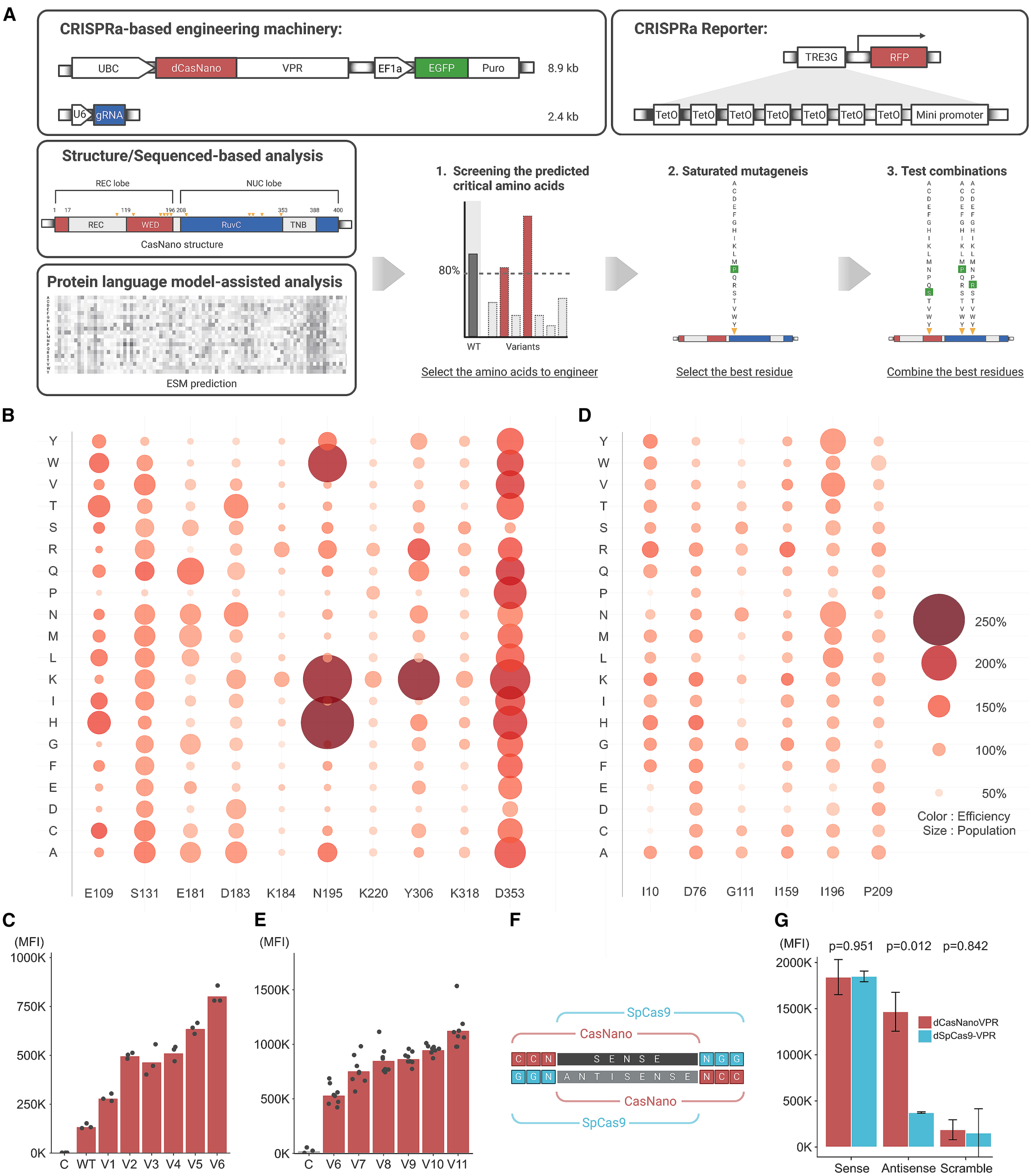

Figure 1. Iterative protein engineering to improve CasNano genome targeting efficiency

(A) Schematics of the expression constructs for dCas-VPR and sgRNA, the TRE3G-RFP reporter integrated into the HEK293T genome, and the iterative protein engineering strategy. (B) Saturated mutagenesis screen of 10 candidate amino acid residues identified from an initial screen. Each residue was mutated to all other 19 amino acids. Bubble plot shows relative RFP activation, measured by flow cytometry and normalized to wild-type dBlCas12f-VPR. Color and size of circles represent efficiency and population of RFP-positive cells, respectively. ($n = 3$) (C) Stepwise improvement of dBlCas12f-VPR activity by combinatorial mutagenesis. Variants V1–V6 were generated by

(legend continued on next page)

levels of endogenous gene activation, comparable to, or exceeding, benchmark SpCas9 for the guide RNAs (gRNAs) tested in this study. CasNano operates with high fidelity and is versatile for precise epigenetic editing. CasNano recognizes a distinct, GC-rich CCN PAM, making it well-suited to target regulatory elements and expand targetable sites in the human genome. In summary, CasNano's minimal size, high efficiency, and target specificity make it a powerful new tool for diverse genome engineering applications. Its compact nature allows for therapeutic strategies requiring AAV or LNP delivery, potentially expanding the target tissues treated with next-generation gene therapies.

RESULTS

Engineering ultracompact Cas12f protein into a potent genome targeting tool

We screened a series of Cas12f family proteins and chose a compact, 400-amino-acid Cas protein identified from *Blautia lenta*, a member of the Cas12f family, hereafter BlCas12f. To characterize its targeting requirements, we tested its activity on matrix of TetO-RFP reporter constructs with 4-digit randomized protospacer adjacent motifs (PAMs) in the 5′ end of TetO. This screen revealed a strong preference for a short, CG-rich PAM, with a near-absolute requirement for cytosine (C) at the -2 and -3 positions relative to the protospacer, while showing flexibility at the -1 and -4 positions (Figure S1). This defines a core 5′-CCN-3′-PAM, which is in stark contrast to the AT-rich PAMs recognized by most Cas12a/f proteins and is complementary to the "NGG" PAM of SpCas9. This unique PAM supports its potential use to target CG-rich regulatory regions such as promoters.

Our goal was to transform BlCas12f into a versatile DNA-targeting platform. We tested the nuclease activity of wild-type BlCas12f with its natural CRISPR RNA (crRNA) and trans-activating crRNA (tracrRNA) but detected almost no activity for double-strand DNA break in mammalian cells (Figure S2B). We then created a nuclease-deactivated (dCas) version for transcriptional regulation by introducing D216A and D390A mutations, on the basis of sequence alignments with catalytically important residues in UnCas12f and AsCas12f (Figure S2A). We fused the dBlCas12f to the VPR transcriptional activator (Figure 1A) and co-expressed with a synthetic single guide RNA (sgRNA), formed by linking its natural crRNA and tracrRNA with a GAAA linker (Figure 2A). To assess its function, we developed a HEK293T reporter cell line with an integrated RFP gene driven by a TRE3G promoter (Figure 1A). The dBlCas12f-VPR and gRNA constructs were delivered by transfection, and RFP expression was quantified by flow cytometry. In this system, the wild-type dBlCas12f-VPR exhibited negligible reporter activation (Figure S3).

To enhance its activity, we initiated an iterative protein engineering campaign directly on this dBlCas12f-VPR backbone (Figure 1A). Using structure-based analysis (specifically, sequence and structural alignments of BlCas12f with other well-characterized Cas12f proteins such as AsCas12f and UnCas12f) alongside a protein language model (the ESM-1b model accessed via the ESM-Scan tool), we identified 22 candidate residues (Table S1) to arginine—a strategy shown to enhance DNA binding in previous engineering efforts of related miniature Cas proteins[14,16–21]—or to variants predicted by the ESM-1b model (https://github.com/facebookresearch/esm).[22] We hypothesized that residues tolerating these initial substitutions without a severe loss of function would represent structurally flexible hotspots ideal for further optimization. While the majority of these initial mutations nearly abolished editing activity, 10 specific residues successfully retained >80% or exhibited higher than wild-type activity. We defined these 10 residues as promising candidates, which were subsequently subjected to saturated mutagenesis to find the optimal substitution for enhancing RFP activation (Figure 1B). The full set of 22 initially screened candidate residues, the substitutions tested, and the corresponding activity in the TRE3G-RFP reporter are provided in Table S1, where the 10 residues advanced to saturated mutagenesis are underlined. Alongside these, we also directly evaluated highly confident specific substitutions predicted by the ESM-1b model, such as T307K, without full saturation. We then combined the most beneficial mutations to leverage synergistic effects. During combinatorial screening, we carefully selected mutations on the basis of their synergistic performance rather than solely on their individual activity. Although N195H showed higher individual activity than N195K (Figure 1B), pairwise combinatorial testing of N195K vs. N195H against each of the nine top second-step mutations revealed that [N195K + Y306K] outperformed [N195H + Y306K] and was the highest-mean fluorescence intensity (MFI) variant among all 22 conditions tested (Figure S3), leading us to select N195K as the V1 scaffold. Starting with the optimal trajectory of V1 (N195K), we progressively added mutations to create V2 (Y306K), V3 (T307K), V4 (S131C), V5 (E181Q), and finally V6 (D183T). This stepwise combination resulted in a ∼600% improvement in activity over the wild-type dBlCas12f (Figure 1C).

Structural comparisons to the Cas12a family identified six additional residues of interest,[23–26] which were subjected to saturated mutagenesis to identify their optimal substitutions (Figure 1D). These beneficial mutations were then added to the V6 backbone to create a new series of variants: V7 (I10R), V8 (I159R), V9 (D76K), V10 (G111N), and V11 (I196V). This doubled the activation potency, with V11 showing the highest activity (Figure 1E). This final, highly active variant was named dCasNano. We confirmed that while the V11 nuclease-active

progressively combining the most effective mutations identified in the screen from (B). (n = 3) (D) Saturated mutagenesis screen of 6 additional amino acid residues identified from structural comparisons with the Cas12a family. Bubble plot shows relative RFP activation, measured by flow cytometry and normalized to dCasNano V6. (n = 3) (E) Further improvement of activity by incorporating the best mutations from the screen in (D) into the V6 backbone, generating variants V7–V11. (n = 7) (F) Schematic of the dual-PAM target site criteria (5′-CCN-N20-NGG-3′) allowing for direct comparison of dCasNano and dSpCas9 using the same spacer sequence on either sense or antisense strands. (G) Direct comparison of the final engineered dCasNano-VPR (V11) and dSpCas9-VPR on the dual-PAM reporter in HEK293T cells. Both systems used the same spacer sequence to target either the sense or antisense strand of the promoter. (n = 3) All data are represented as mean ± SD. See also Figures S1–S3; Table S1.

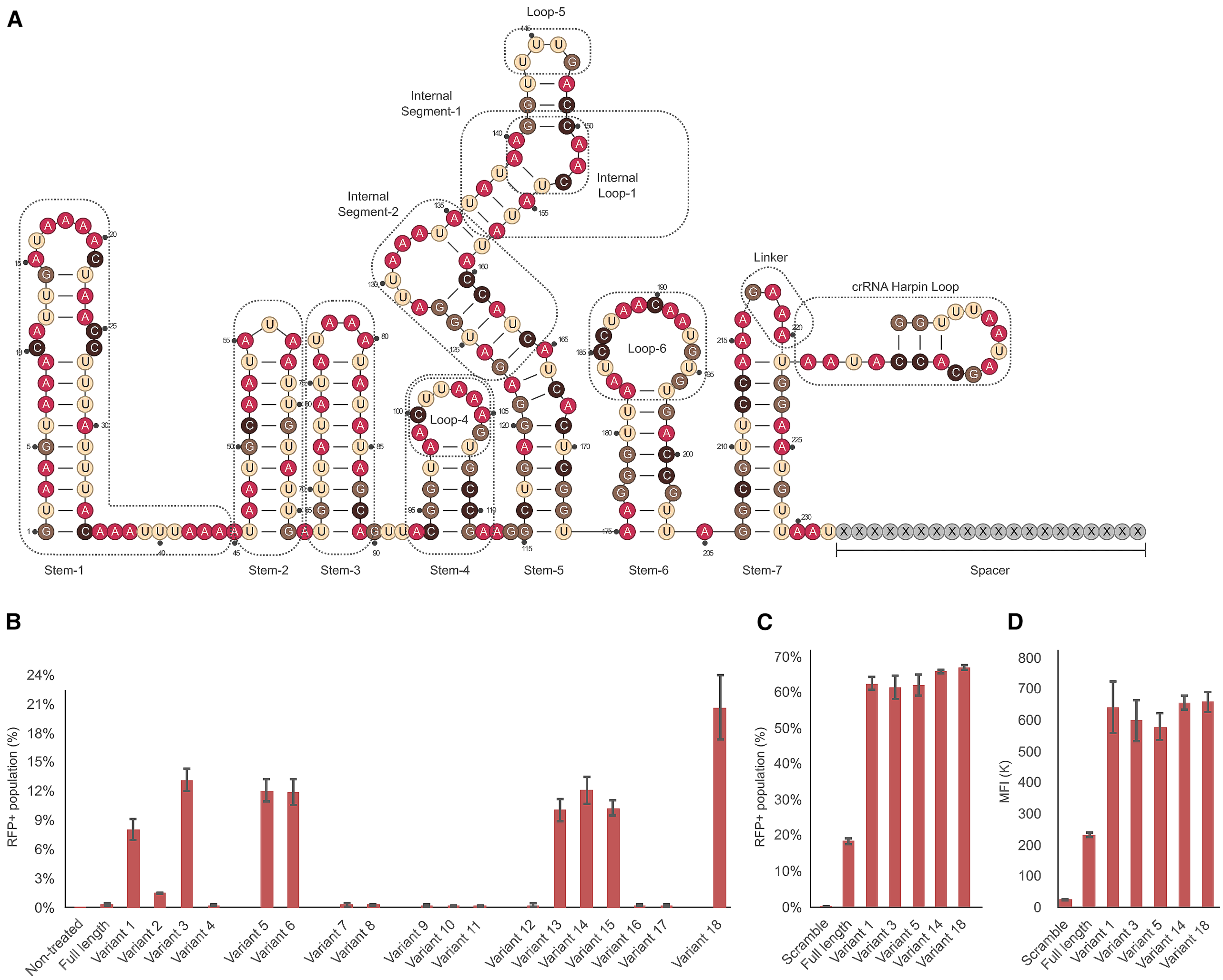

Figure 2. Systematic engineering of the sgRNA scaffold boosts CasNano activity

(A) Predicted secondary structure of the initial 233-nt sgRNA, created by linking the natural tracrRNA and crRNA of BlCas12f (r.216 to r.221) by a GAAA linker. Key structural domains are indicated. (B) Screen of 18 sgRNA variants with systematic truncations and modifications. Bar chart shows the percentage of RFP-positive cells for each variant co-expressed with wild-type dBlCas12f-VPR. (C and D) Comparison of the most effective sgRNA variants (1, 3, 5, 14, and 18) when paired with the fully engineered dCasNano-VPR (V11). Bar charts show the percentage of RFP-positive cells (C) and the mean fluorescence intensity (MFI) (D). All data are represented as mean ± SD for $n = 3$ biological replicates. See also Table S3.

backbone, when paired with the fully optimized sgRNA scaffold, regained only weak cleavage activity, the D216A and D390A mutations in dCasNano completely abolished it (Figure S2B).

Finally, we benchmarked the engineered dCasNano-VPR against dSpCas9-VPR. We designed the TRE3G reporter to contain both a CCN PAM for dCasNano and an NGG PAM for dSpCas9 flanking the TetO sequences. This dual-PAM design allows both effectors to target the same protospacer on either the sense or antisense strand (Figure 1F). When targeting the sense strand, dCasNano-VPR induced reporter activation at levels comparable to dSpCas9-VPR. When targeting the antisense strand, dCasNano-VPR outperformed dSpCas9-VPR (~4-fold higher MFI), demonstrating robust and strand-independent activity (Figure 1G). Because intracellular editor expression and active-complex abundance were not measured, all comparisons between dCasNano and dSpCas9 reported here represent functional outputs under equal-mass plasmid transfection for the specific gRNAs and loci tested and do not distinguish intrinsic editor activity from differences in plasmid delivery, expression, or complex assembly. Thus, our engineering strategy transformed the native BlCas12f protein into a highly efficient genome targeting tool.

Systematic engineering of the sgRNA scaffold to decrease length and boost dCasNano activity

In parallel with protein engineering, we optimized the sgRNA scaffold to decrease length and enhance system performance. The initial 233-nt

sgRNA used in our screens was created by removing the natural crRNA hairpin loop and joining the crRNA and tracrRNA with a GAAA linker. The predicted secondary structure of this sgRNA is highly complex (Figure 2A), which we hypothesized it might be suboptimal for ribonucleoprotein formation and function in mammalian cells.

To identify a more effective and compact sgRNA architecture, we pursued a multi-step engineering strategy by using the wild-type dBlCas12f-VPR. We first systematically trimmed the 5′ end of the tracrRNA and generated variant 1 (Δstem-1 del r.1–45), variant 2 (Δstem-1 to 2; del r.1–67), variant 3 (Δstem-1 to 3; del r.1–94), and variant 4 (Δstem- 1 to 4; del 1–114), which revealed that removing stems 1–3 (variant 3) yielded the highest activity while shortening the scaffold to 140 nt (Figure 2B). Using this improved variant 3 as a backbone, we explored further modifications. While abridging the linker of variant 5 (del r.214–221 ins GAAA) and variant 6 (del r.214–221 ins UUCG) were tolerated, we found many structural elements were essential for function, as modifying 3′ tail in variant 7 (r.228–229UU>CC) and variant 8 (del r.230–232) or truncating internal segments in variant 9 (Δinternal segment-1; del 137–141; 150–157), variant 10 (Δinternal loop-1; del 139–141; 150–154), and variant 11 (Δinternal segment-2; del 123–136; 158–164) abolished activity.

Modifying loop-4 and loop-6 in variant 12 (del r.99–102) or variant 16 (del r.183–196 ins GAAA) and variant 17 (del r.183–196 ins UUCG) also nullified the editing activity. We identified modifications in loop-5 that were well tolerated and prevented the formation of a problematic poly-uridine stretch in variant 13 (del r.144–147 ins GAAA), variant 14 (del r.144–147 ins UUCG), and variant 15 (del r.143–148 ins GUUCGC). By combining the tolerated linker from variant 5 with the beneficial loop-5 modification from variant 14, we created a final combination variant (variant 18) that outperformed all previous designs.

We then tested the most promising designs (variants 1, 3, 5, 14, and 18) with the final engineered dCasNano-VPR (V11). While variant 18 remained the top performer, the efficiency gains over these engineered variants were less pronounced when paired with the highly active protein (Figures 2C and 2D). Given its superior activity and compact 136-nt length, the variant 18 gRNA scaffold was selected for all subsequent experiments.

Engineered dCasNano demonstrates high specificity and low tolerance to mismatches

To attain high specificity for genome engineering, we comprehensively evaluated the targeting fidelity of dCasNano-VPR by assessing its sensitivity to guide-target mismatches and by profiling its global off-target effects on the transcriptome. We generated a library of sgRNAs containing single nucleotide mismatches at each position from 1 to 20 of the protospacer targeting our RFP reporter (Figure 3A). At each position, a single transversion-type substitution was applied following the rule C↔A and G↔T (i.e., C→A, A→C, G→T, and T→G). These transversions generate same-class mismatches at the substituted position—purine:purine (A:G) and pyrimidine:pyrimidine (T:C)—that perturb both Watson-Crick base pairing geometry and minor-groove hydrogen bonding, providing a stringent probe of guide-target specificity. When tested in the TRE3G-RFP reporter cell line, dCasNano-VPR exhibited exquisite sensitivity to mismatches. It outperformed dSpCas9-VPR when mismatches were located in positions 1–15, with particularly high sensitivity in the 1–7 seed region. SpCas9, conversely, showed greater sensitivity only at position 8 (Figure 3B).

To assess genome-wide specificity in an unbiased manner, we performed RNA sequencing (RNA-seq) on HEK293T reporter cells expressing either dCasNano-VPR or dSpCas9-VPR with a targeting or a non-targeting scrambled sgRNA. We utilized this genomically integrated reporter system to assess editor specificity, as activating an endogenous functional gene can trigger downstream biological networks that confound the identification of true, direct off-target editing events.[14] Both systems strongly upregulated the RFP target (Figures 3C–3E). While the absolute expression level of RFP was higher with dSpCas9-VPR, dCasNano-VPR achieved a greater fold-change activation relative to its scramble control (∼394-fold for dCasNano-VPR vs. ∼128-fold for dSpCas9-VPR), corresponding to a higher signal-to-background ratio in this assay. A global comparison of gene expression profiles revealed that both dCasNano-VPR and dSpCas9-VPR induced minimal off-target transcriptional changes, with the vast majority of transcripts remaining close to the diagonal in the scatterplot analyses. To quantify this, we analyzed the standard deviation of expression for all genes across the targeting and non-targeting replicates. The resulting distributions for dCasNano and dSpCas9 were narrow and had similar medians, confirming that both systems have a minimal off-target profile at the whole-transcriptome level (Figure 3F). To specifically test whether dCasNano-VPR might engage genomic loci with partial sequence similarity to the on-target protospacer, we identified candidate off-target sites within four mismatches of the protospacer with Cas-OFFinder. Because the two editors recognize PAMs on the opposite sides of the protospacer, searches were performed separately for each system, which yielded largely distinct candidate sets: 28 expressed genes for dCasNano-VPR and 38 for dSpCas9-VPR, of which only four were shared. No genomic site within two mismatches of the protospacer was identified for either editor, and the closest candidates carried three mismatches (three genes for dCasNano-VPR and four for dSpCas9-VPR). Neither editor systematically activated its own predicted off-target genes, and the log2 fold-change distributions of the two off-target sets were indistinguishable from each other (Kolmogorov-Smirnov $p = 0.43$; Mann-Whitney $p = 0.78$) and from the genome-wide background (Figure S4). At the four loci predicted as off-target candidates for both editors, neither system showed activation. Together, these results demonstrate that dCasNano possesses high genome-wide targeting specificity, with a fidelity profile that is similar or superior to dSpCas9 system for the gRNAs tested in this study.

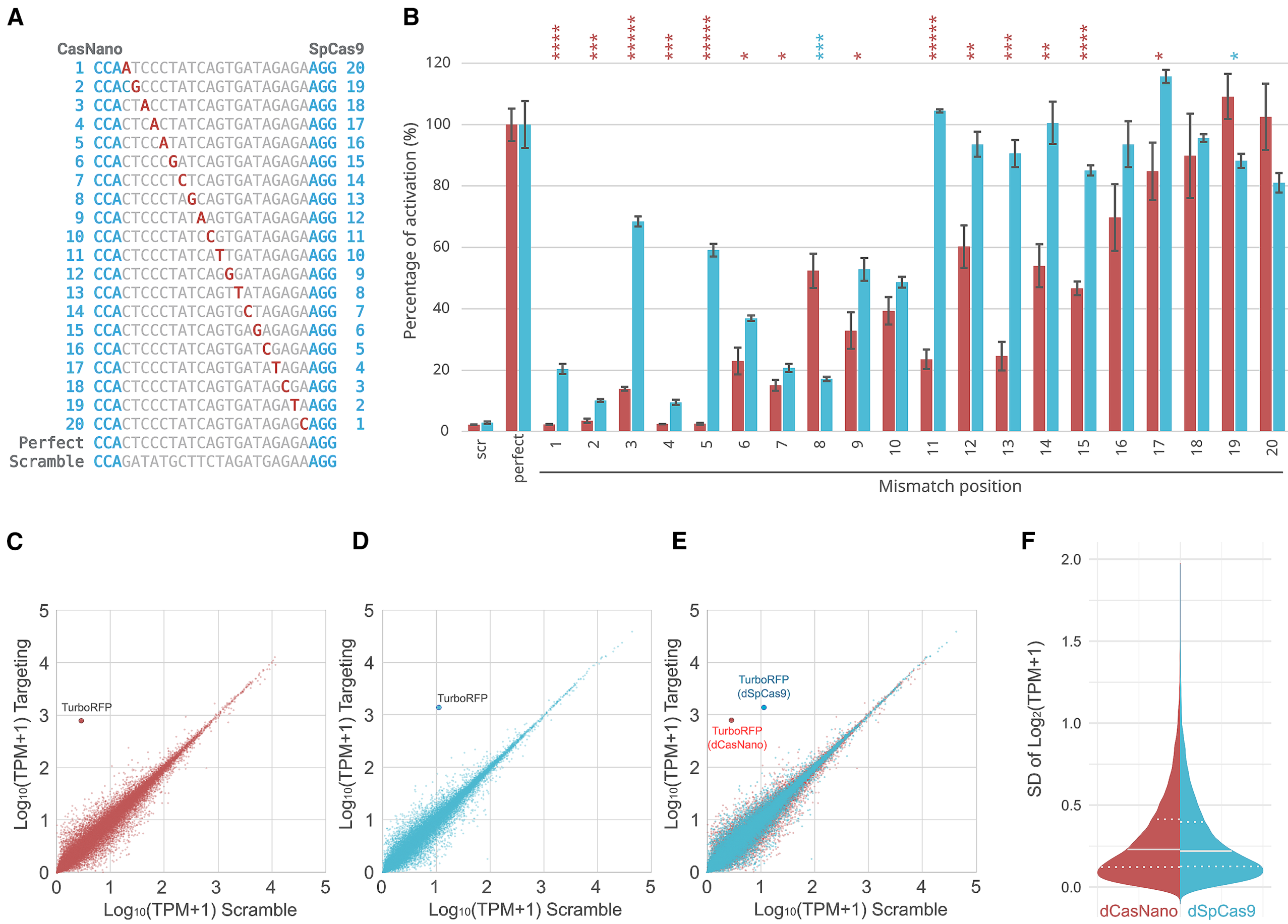

Figure 3. Evaluation of CasNano specificity

(A) Schematic of the sgRNA library used to test mismatch sensitivity, containing single nucleotide substitutions at each position of the 20-nt protospacer. Positions are numbered 1–20, with position 1 being the nucleotide proximal to the PAM of each Cas system. (B) Comparison of mismatch sensitivity between dCasNano-VPR (red) and dSpCas9-VPR (light blue) for TRE3G-RFP reporter activation. The activity of each system with a perfectly matched sgRNA is set to 100%. (C–E) Scatterplots of whole-transcriptome expression profiles comparing dCasNano-VPR (C) or dSpCas9-VPR (D) with a targeting sgRNA to a non-targeting (scrambled) sgRNA. Gene expression is shown as Log10(TPM+1). The on-target RFP transcript is indicated. Data shown are the mean of two biological replicates. (F) Violin plots showing the distribution of standard deviations for all expressed genes across targeting and non-targeting replicates for each system, quantifying global off-target transcriptional effects. *$p < 0.05$; **$p < 0.01$; ***$p < 0.001$; ****$p < 0.0001$; *****$p < 0.00001$. See also Figure S4.

Activation of endogenous genes with the dCasNano-VPR system

To validate the efficacy of dCasNano-VPR on endogenous targets, we next sought to activate gene expression in HEK293T cells and directly compare its performance to dSpCas9-VPR. We selected two target genes, *HBG1* and *IL1RN*, and designed pools of 4 sgRNAs targeting their respective promoter regions (Figures 4A and 4B). We specifically selected target sites compatible with both systems to enable a direct comparison by using the same spacer sequences for both sense and antisense strands.

In HEK293T cells, dCasNano-VPR activated both target genes after a 72 h incubation. For *HBG1*, dCasNano-VPR induced potent activation from sense and antisense strands at similar levels as dSpCas9-VPR (Figure 4C). When targeting the *IL1RN* promoter, activation levels were again similar between the two systems for the sense strand. Notably, when targeting the antisense strand of *IL1RN*, dCasNano-VPR was significantly more effective than dSpCas9-VPR (~3.3-fold greater activation, p = 0.010) (Figure 4D). These results confirm that dCasNano-VPR is a highly effective activator of endogenous human genes, with a potency comparable or superior to the dSpCas9 system under equal-mass plasmid transfection for the gRNAs tested in this study.

Targeted epigenetic silencing with a DNMT3A-dCasNano fusion

To demonstrate the utility of dCasNano beyond transcriptional activation, we repurposed it as a platform for targeted epigenetic modification. We fused dCasNano to the catalytic domain of the DNA methyltransferase DNMT3A and evaluated its ability to induce targeted gene silencing. For this, we first utilized a previously reported

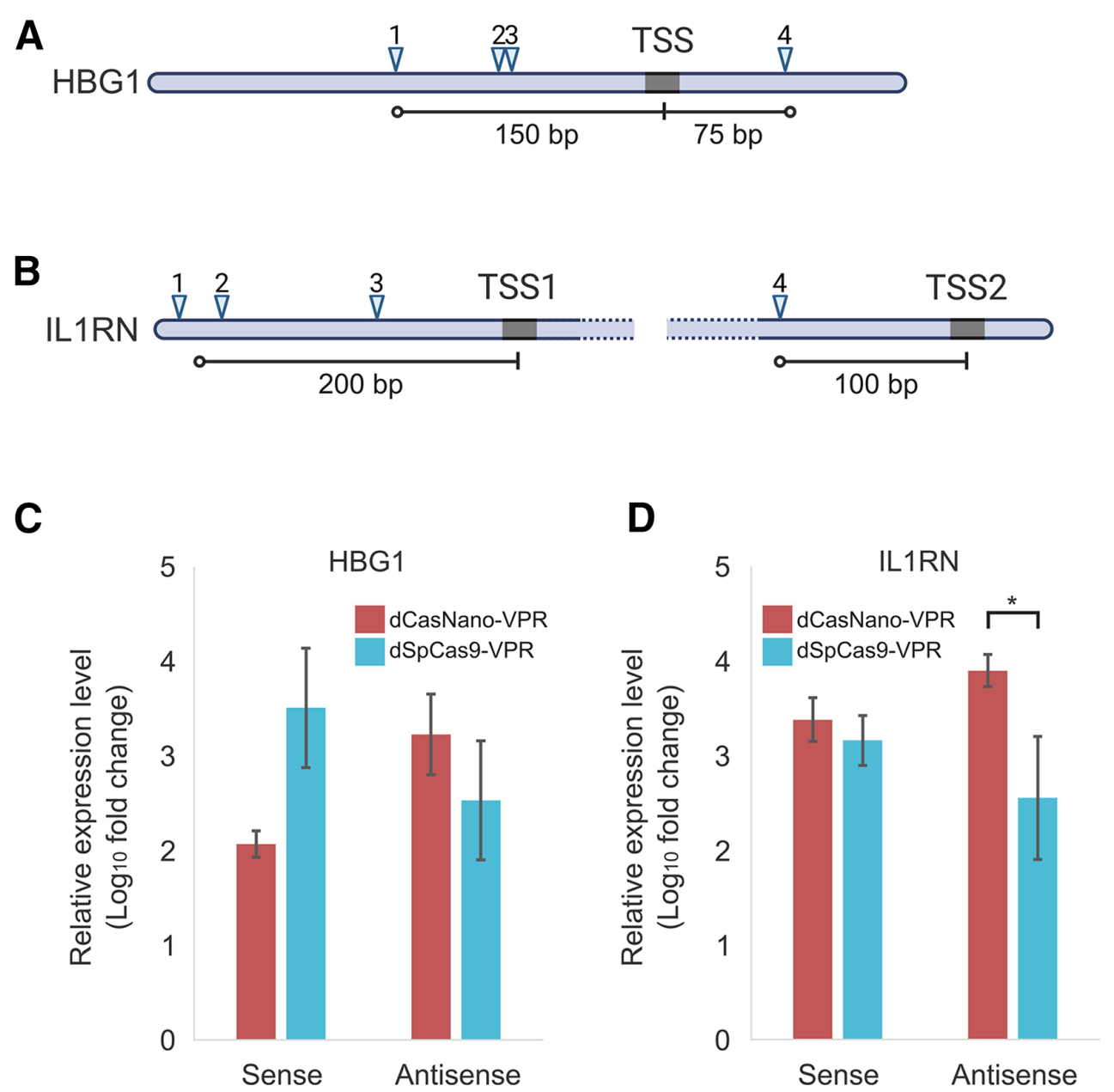

Figure 4. Activation of endogenous *HBG1* and *IL1RN* genes in HEK293T with the dCasNano-VPR

(A and B) Schematics of the human *HBG1* (A) and *IL1RN* (B) promoter regions showing the locations of sgRNA target sites on both sense and antisense strands. (C and D) Relative expression levels of *HBG1* (C) and *IL1RN* (D) in HEK293T cells determined by qPCR at 72 h after transfection with dCasNano-VPR or dSpCas9-VPR and pools of 4 sgRNAs targeting either the sense or antisense strand. Data are shown as Log10-fold change relative to a non-targeting control. Data are represented as geometric mean ± SD for n = 3 biological replicates. Statistical significance was determined by a two-tailed Student's t test. *$p < 0.05$. See also Table S4.

HEK293T reporter cell line containing a genome-integrated GAPDH-Snrpn-EGFP cassette, in which targeted methylation of the promoter region leads to a decrease in EGFP expression (Figure S5A).[27] Flow cytometry analysis revealed that DNMT3A-dCasNano induced robust silencing, with a nearly 25% EGFP-negative population after 6 days of incubation, which was comparable to the silencing achieved by DNMT3A-dSpCas9 (Figure 5A). This corresponded with a marked decrease in the MFI of the EGFP signal, which was also comparable to the dSpCas9-based editor (Figure 5B). To confirm that the observed silencing was a direct result of DNA methylation, we performed bisulfite sequencing on the targeted reporter promoter. This analysis showed that DNMT3A-dCasNano induced methylation across the targeted CpG sites, with levels similar to or exceeding those induced by DNMT3A-dSpCas9 (Figures 5C and 5D).

To test the system on an endogenous gene, we targeted the promoter of MECP2 in standard HEK293T cells, which possess three active and one inactive X chromosome.[28] To ensure a rigorously fair comparison and definitively exclude any locus-dependent targeting bias, we explicitly targeted the exact same genomic loci for both effectors by designing gRNAs that engage opposite strands within the same target windows. Bisulfite sequencing revealed that while the basal methylation level was low, DNMT3A-dCasNano induced an increase in methylation at the targeted locus. Strikingly, under the same conditions, DNMT3A-dSpCas9 induced lower methylation at the endogenous MECP2 locus (Figure 5E). These results establish dCasNano as a compact and highly efficient platform for targeted epigenetic silencing, further highlighting its versatility and robust activity at the endogenous loci tested in this study.

To assess whether DNMT3A-dCasNano-deposited methylation persists after the editor is no longer expressed, we extended the GAPDH-Snrpn-EGFP reporter experiment to 2 weeks post-transfection and profiled methylation at 29 anchor CpG positions across the targeted Snrpn promoter by bisulfite sequencing. At this time point, methylation in the DNMT3A-dCasNano targeting condition had returned to within the range of the matched scramble, mock, and non-treated controls; the same was observed for DNMT3A-dSpCas9 (Figure S7). Durable silencing was, therefore, not demonstrated for either DNMT3A editor in this reporter system under the conditions tested.

Targeted demethylation and gene reactivation in HEK293T and hESCs

To assess the versatility of the CasNano platform for bidirectional epigenetic editing, we engineered a TET1-dCasNano fusion protein for targeted DNA demethylation. We first tested its ability to reactivate a previously reported DAZL-Snrpn-mCherry reporter cell line, where the mCherry cassette is silenced by CpG methylation spreading from the neighboring, heavily methylated DAZL promoter (Figure S5B).[27] Upon transfection with TET1-dCasNano and sgRNAs targeting the Snrpn promoter, we observed a reactivation of mCherry expression, reaching levels comparable to those achieved with a TET1-dSpCas9 fusion protein (Figure 6A). To confirm that this reactivation was due to epigenetic modification, we conducted bisulfite sequencing on the targeted Snrpn promoter. The analysis revealed a decrease in methylation levels at the targeted CpG sites for cells treated with TET1-dCasNano, consistent with the observed gene reactivation (Figure 6B). The demethylation efficiency was comparable to that of TET1-dSpCas9, with both editors reducing the median methylation levels significantly compared to scrambled gRNA controls (Figure 6C).

To assess this system in a therapeutically relevant context, we applied TET1-dCasNano to reactivate a silenced endogenous gene in a previously described human embryonic stem cell (hESC) reporter line (29-G) (Figure S5C).[29] In these cells, a tdTomato reporter is fused in-frame with the end of exon 3 on one allele of the MECP2 gene, which is subject to X chromosome inactivation and is silenced by DNA methylation. After 6 days of transfection with the editors and sgRNAs targeting the silenced MECP2 promoter, both TET1-dCasNano and TET1-dSpCas9 induced reactivation of the tdTomato reporter, achieving comparable percentages of tdTomato-positive cells (Figure 6D). However, the TET1-dCasNano-treated cells exhibited a notably higher MFI, suggesting higher per-cell reactivation (Figure 6E). While not confirmed, this difference in MFI may be

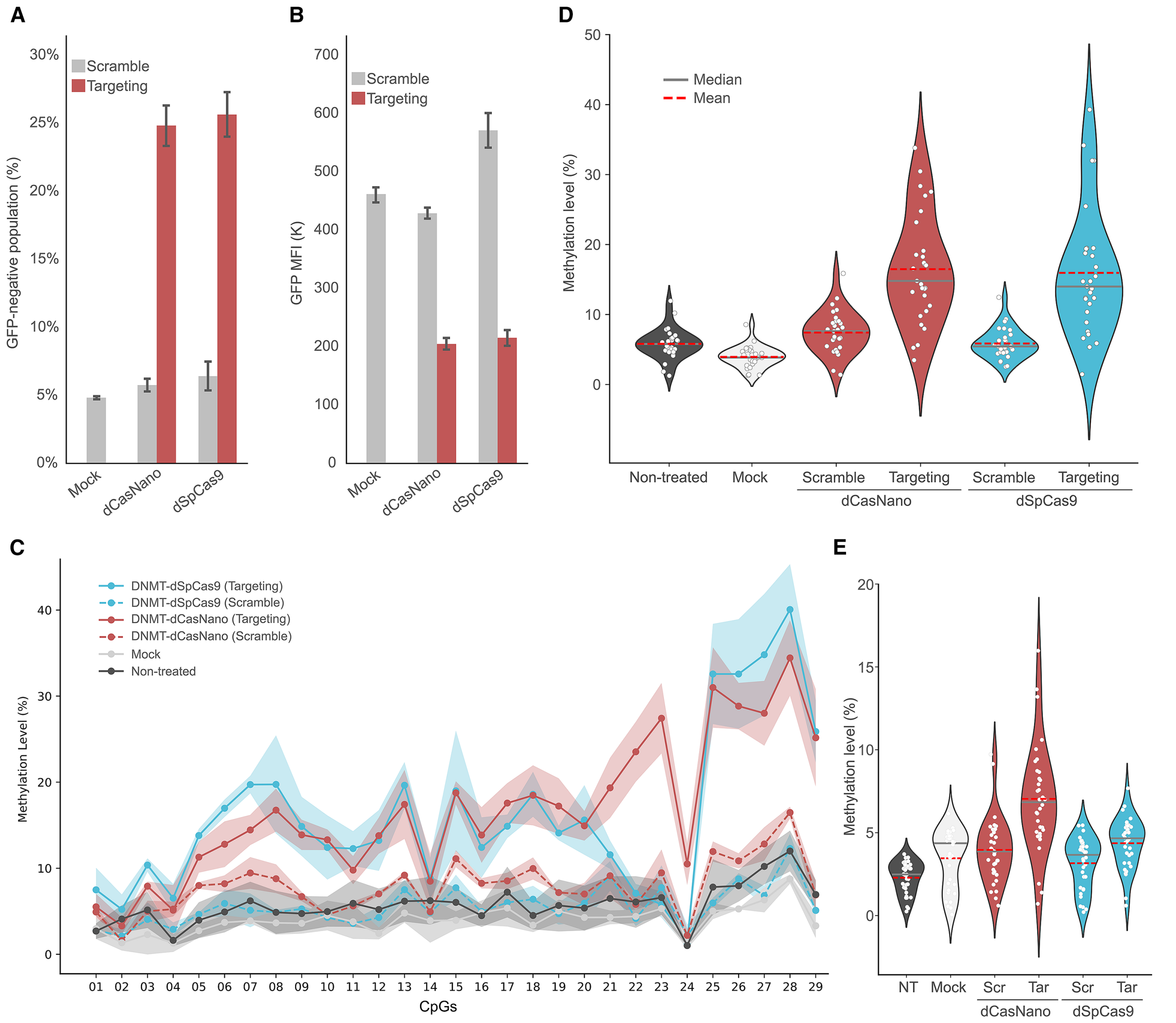

Figure 5. Targeted epigenetic silencing using a DNMT3A-dCasNano fusion

(A and B) Quantification of gene silencing in a HEK293T GAPDH-Snrpn-EGFP reporter cell line. Bar charts show the percentage of EGFP-negative cells (A) and the mean fluorescence intensity (MFI) of EGFP (B) at 6 days after transfection with DNMT3A-dCasNano or DNMT3A-dSpCas9 plasmids. (C) DNA methylation levels at individual CpG sites within the targeted GAPDH-Snrpn promoter region in the reporter cell line, as measured by bisulfite sequencing. The shadow represents SD. (D) Violin plots summarizing the distribution of methylation levels from (C) across all targeted CpG sites for the GAPDH-Snrpn reporter. (E) Violin plots showing the distribution of methylation levels at the endogenous MECP2 promoter in standard HEK293T cells following treatment with the indicated editors. NT, non-treated; Scr, scramble gRNA; Tar, targeting gRNA. All data are represented as mean ± SD for n = 3 biological replicates. See also Figures S5 and S7.

attributable to more efficient delivery of the smaller TET1-dCasNano plasmid compared to its larger dSpCas9 counterpart. A defining feature of therapeutic epigenome editing is the long-term durability of the induced transcriptional changes. To evaluate the persistence of CasNano-mediated epigenetic reprogramming, we monitored the TET1-dCasNano-edited 29-G hESCs over an extended period. Remarkably, the reactivation of the silenced MECP2 allele was stably maintained for up to 5 months post-transfection. Both the percentage of tdTomato-positive cells and the overall expression levels closely mirrored the positive control line throughout the entire 5-months duration (Figure S6). Bisulfite sequencing at the 5-month time point further confirmed persistent loss of CpG methylation at the targeted MECP2 promoter (Figures S6C and S6D).

DISCUSSION

We engineered a novel ultracompact Cas12f effector, CasNano, into a potent and versatile platform for mammalian genome engineering.

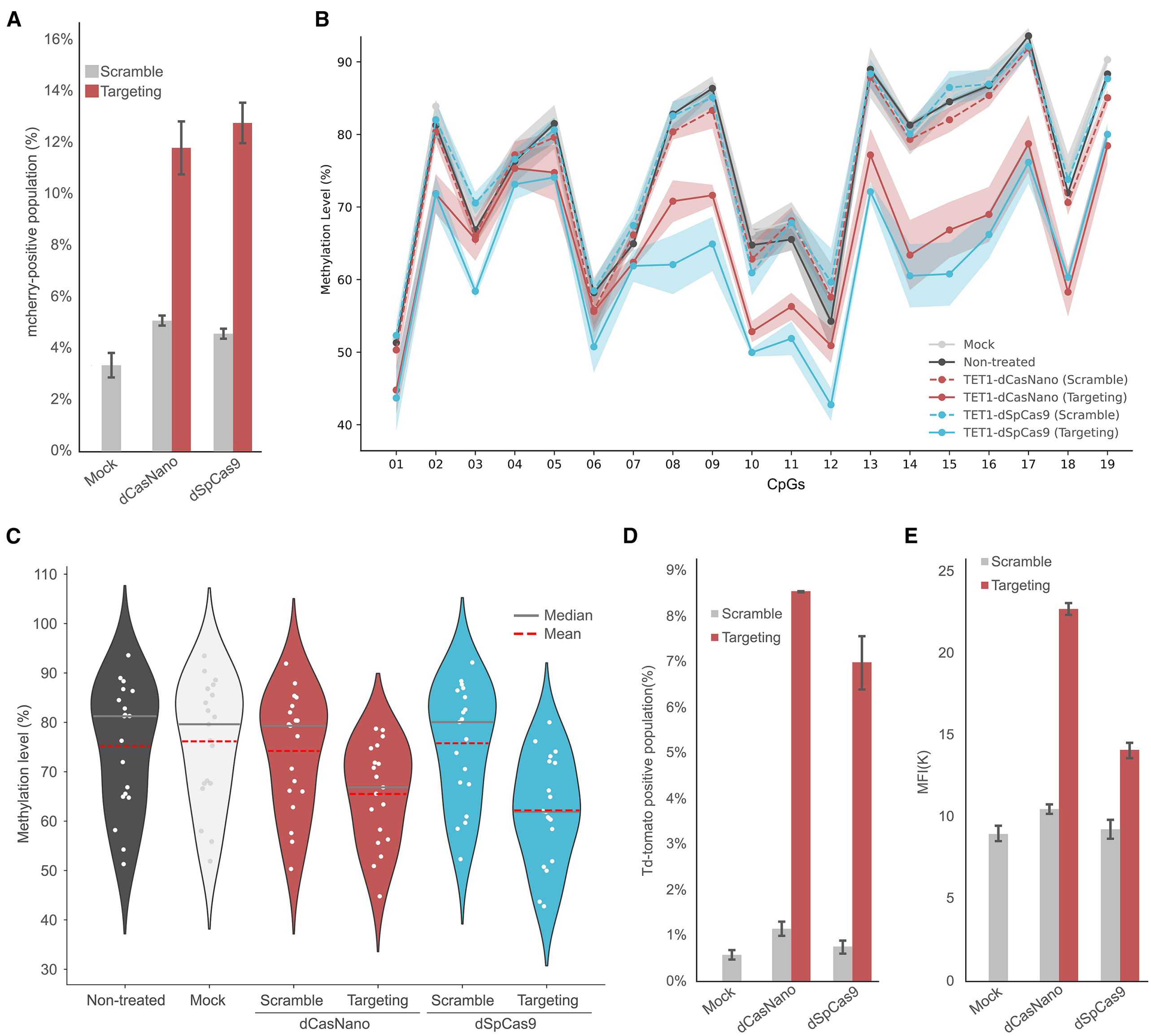

Figure 6. Targeted demethylation and reactivation of a silenced reporter using TET1-dCasNano

(A) Quantification of gene reactivation in a HEK293T DAZL-Snrpn-mCherry reporter cell line. The bar chart shows the percentage of mCherry-positive cells at 6 days after transfection with TET1-dCasNano or TET1-dSpCas9 plasmids. (B) DNA methylation levels at individual CpG sites within the targeted Snrpn promoter region, as measured by bisulfite sequencing. The shaded area represents the standard deviation (SD). (C) Violin plots showing the distribution of methylation levels across all targeted CpG sites for each condition, comparing the median (solid line) and mean (red dashed line) methylation levels. (D and E) Quantification of endogenous *MECP2* gene reactivation in 29-G hESC reporter line. Bar charts show the percentage of tdTomato-positive cells (D) and the mean fluorescence intensity (MFI) of tdTomato (E) at 6 days after transfection with TET1-dCasNano or TET1-dSpCas9 plasmids. All data are represented as mean ± SD for $n = 4$ biological replicates. See also Figures S5 and S6.

Our work transforms a naturally occurring protein with negligible activity into a robust tool for transcriptional activation, targeted methylation, and targeted demethylation, with similar or superior efficacy, for the gRNAs and loci tested in this study, compared to the benchmark SpCas9 with a large protein size.

Our protein engineering strategy yielded several key insights. While many Cas engineering efforts focus on substituting key residues with arginine to enhance DNA binding, our saturated mutagenesis screens revealed this was not always the optimal choice for BlCas12f. Substitutions to other positively charged residues (i.e., lysine or histidine), or to uncharged residues, conferred superior activity. Thus, enhancing a miniature Cas protein's function may require optimization of stereochemistry and charge distribution at the DNA-binding interface rather than simply increasing positive charge.

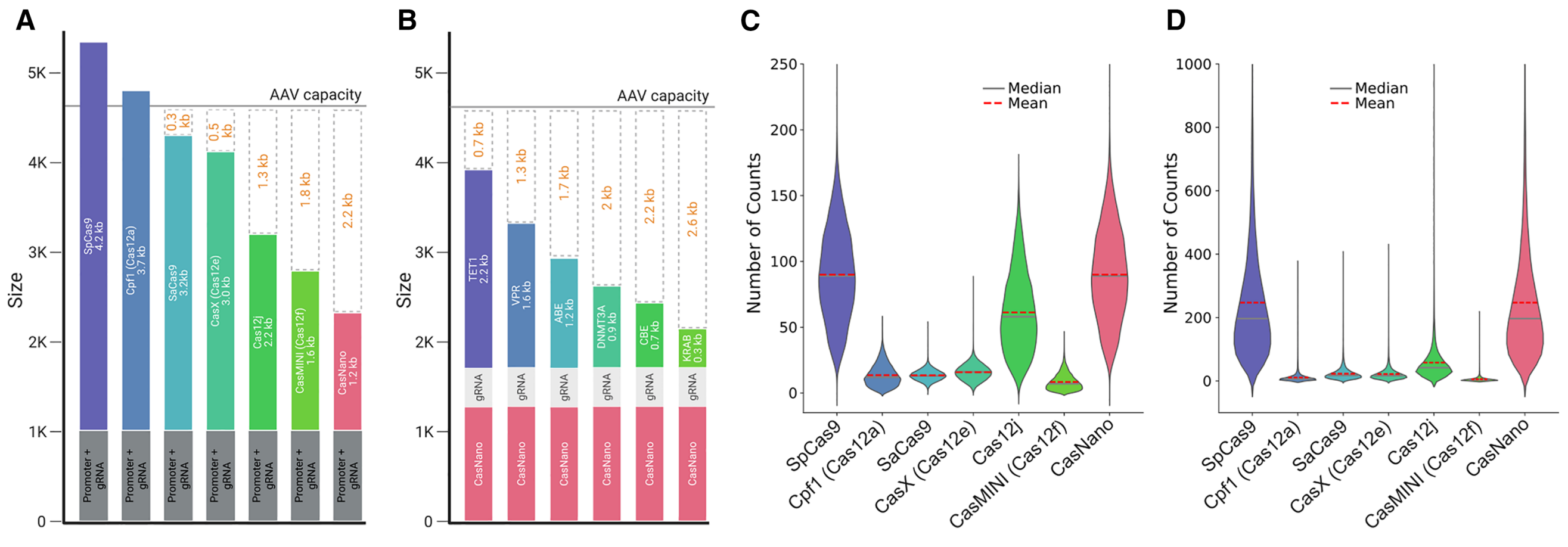

Figure 7. Compact size and flexible PAM facilitate broad CasNano applicability

(A) Bar chart comparing the remaining AAV packaging capacity available for an effector domain after including the coding sequences for various Cas proteins. Calculation assumes a 1 kb allowance for the promoter and sgRNA expression cassette within the standard 4.7 kb AAV packaging limit. (B) Bar chart illustrating the remaining AAV cargo space after packaging dCasNano, an sgRNA cassette, and various effector domains (e.g., VPR, KRAB, and TET1), highlighting the capacity for additional components like promoters, tags, or other transgenes. (C) Violin plots showing the distribution of the number of targetable sites for various Cas systems within the 500 bp region immediately upstream of the transcription start site (TSS) for 19,477 human genes. (D) Violin plots showing the distribution of targetable sites within the CpG islands of the regulatory regions of 12,348 human genes, comparing CasNano to other major Cas systems.

In parallel, our systematic sgRNA engineering reduced the scaffold length to 136 nt and enhanced activity. This compact sgRNA is advantageous for AAV delivery where cargo capacity is limited. While our scaffold is longer than SpCas9 and the engineered CasMINI ge3.1, its high activity and small size represent a significant step forward.[30] Future work could further reduce the size and increase the efficacy, although our data suggest that many core tracrRNA elements are functionally essential. The long-term stability of this engineered sgRNA *in vivo* warrants further investigation.

Two therapeutic advantages of CasNano are its ultracompact size and its flexible, GC-rich PAM sequence, which overcome major limitations of large and miniature CRISPR systems. AAV capsids cannot deliver large, multi-component CRISPR systems. Many Cas effectors (e.g., SpCas9, SaCas9, Cpf1/Cas12a, and CasX/Cas12e) consume most of the ∼4.7 kb cargo capacity, leaving insufficient space for effector domains (Figure 7A). By contrast, the tiny CasNano (∼1.2 kb) provides packaging flexibility and can be combined with a diverse range of effectors (e.g., base editors [ABE/CBE], transcriptional regulators [VPR/KRAB], and epigenetic modifiers [TET1/DNMT3A]) while leaving substantial room for promoters, UTRs, multiplexed sgRNA cassettes, or even additional transgenes within a single AAV (Figure 7B). While our *in silico* modeling indicates CasNano's compatibility with single-AAV packaging limits even when fused to large epigenetic effectors, future empirical *in vivo* studies will be essential to fully validate its therapeutic delivery efficiency and biodistribution in animal models. Beyond its size, CasNano's targeting scope is improved over Cas systems constrained by restrictive AT-rich PAMs (e.g., CasMINI/Cas12f's TTTR, Cpf1/Cas12a's TTTV, SaCas9's NGGRRT, or CasX/Cas12e's TTCN), CasNano's simple CCN PAM grants it broad targeting access across the genome. Our bioinformatic analysis of the 500 bp upstream of the transcription start site (TSS) for 19,477 human genes showed that CasNano has a high density of potential target sites, averaging 89.9, comparable to SpCas9 but far higher than those of Cpf1/Cas12a (13.5), CasX/Cas12e (15.7), CasMINI/Cas12f (8.3), or Cas12j (61.2) (Figure 7C). This advantage is more pronounced in epigenetic editing, where targeting GC-rich CpG islands in regulatory regions is paramount. In an analysis of 12,348 genes with promoter-associated CpG islands, CasNano and SpCas9 had an average of 247.3 targetable sites, far exceeding those of Cas12j (58.1), SaCas9 (23.1), CasX/Cas12e (22.0), Cpf1/Cas12a (10.9), and CasMINI/Cas12f (5.6) (Figure 7D). While other miniature Cas12f systems (e.g., UnCas12f or AsCas12f) are ideal size-matched comparators, their strict requirement for highly AT-rich PAMs makes head-to-head functional comparisons at these GC-dense regulatory regions practically impossible. This inherent biological constraint is precisely why we selected SpCas9 as our primary functional benchmark. For instance, within the *MECP2* promoter, there are 120 eligible target sites for CasNano but only 5 theoretical sites for UnCas12f (CasMINI). After excluding sequences with suboptimal complexity or potential off-target binding sites, finding a shared, highly effective spacer sequence for both editors becomes fundamentally unfeasible. Rather than a limitation of our study, this discrepancy perfectly highlights CasNano's core advantage: it unlocks GC-rich regulatory elements that are largely inaccessible to other miniature, AT-restricted Cas systems. CasNano's packaging efficiency and dense targeting in regulatory regions creates a versatile tool for programmable genome and epigenome targeting.

Several methodological considerations underlie our comparative benchmarking of CasNano against SpCas9. The two expression

plasmids differ in coding-sequence length, and the two effectors have fundamentally different stoichiometries—CasNano (Cas12f family) assembles as a homodimer on the gRNA to form the active effector complex, whereas SpCas9 functions as a monomer. Normalization between these two systems can, therefore, be approached along several axes (equal DNA mass, equimolar plasmid copy number, or matching at the transcript or protein level), but no single axis can simultaneously equalize plasmid mass, copy number, transcript level, monomer protein level, and active-complex number across the two platforms. For example, equimolar plasmid delivery—while equalizing input plasmid count—would yield at least 2-fold fewer functional CasNano effector complexes than SpCas9, because two CasNano monomers must dimerize to form a single active unit; this disparity is further amplified by the equilibrium nature of homodimer assembly, in which active-dimer count scales nonlinearly with monomer concentration. We adopted equal-DNA-mass transfection throughout this study, consistent with the convention established in prior comparative studies of compact Cas systems (CasMINI vs. Cas12a[14] and CasX vs. SpCas9[13]). Beyond this methodological precedent, equal-mass transfection offers several additional advantages for comparative editor benchmarking. It constitutes a dose-matched payload comparison, mirroring the *in vivo* scenario, in which a fixed quantity of nucleic acid is administered and editors must compete on per-mass functional output—arguably the most translationally relevant axis for evaluating candidate therapeutic platforms. It also holds the DNA-to-lipid ratio constant across conditions, ensuring that transfection chemistry (lipoplex formation, particle size, and endosomal escape) does not itself become a confounding variable in the comparison. Importantly, we did not quantify intracellular editor expression or active-complex abundance in this study. The comparisons reported here, therefore, describe functional outputs obtained under equal-mass plasmid transfection for the specific gRNAs and loci tested and cannot distinguish intrinsic editor activity from differences in plasmid delivery, expression, or complex assembly. Establishing intrinsic per-complex activity would require direct measurement of editor transcript, protein, and assembled-complex levels, which remains an important direction for future work.

The dimeric architecture of CasNano may also have a functional consequence for effector-fusion applications. Because two CasNano monomers assemble on an sgRNA, a fusion protein is expected to deliver two effector copies to each bound target site, whereas monomeric SpCas9 delivers one. Transcriptional output scales with the number of activation domains recruited to a promoter, so this difference in effector valency could contribute to the activation levels observed here; a similar consideration may apply to the DNMT3A and TET1 fusions. This possibility was not tested in the present study and would require, for example, comparing complexes in which only one of the two monomers carries the effector domain. If confirmed, effector valency would represent an architectural property of the Cas12f scaffold rather than a difference in the intrinsic activity of the DNA-binding module.

Our investigation into the nuclease activity of CasNano revealed that even the fully optimized system exhibits a genome editing efficiency lower than that of SpCas9 (Figure S2B). We propose that this apparent divergence between high CRISPRa efficiency and low nuclease activity stems from the mechanistic decoupling of DNA binding and DNA cleavage. Transcriptional activation relies predominantly on robust target recognition and stable effector engagement at the target locus. Our protein engineering campaign successfully enhanced this DNA-binding capacity. In contrast, indel formation requires not only stable binding but also highly efficient target cleavage and subsequent DNA repair. This is likely due to our engineering strategy, which utilized transcriptional activation as the sole functional readout, thereby optimizing CasNano exclusively for DNA binding and target engagement rather than DNA cleavage. Furthermore, unlike SpCas9 which generates blunt ends, it has been reported Cas12f proteins produce staggered ends on DNA, which are repaired much less efficiently by error-prone non-homologous end joining.[31] Consequently, CasNano acts as an exceptionally strong DNA-binder with naturally constrained cleavage efficiency. Different mutations would be required to enhance the catalytic activity of the RuvC domain for efficient double-strand breaks. Further, like other Cas12f proteins, CasNano has a single RuvC domain and is predicted to function as a homodimer. This architecture increases its difficulty to function as a DNA nickase. Hence, using CasNano for base or prime editing strategies requires further optimization. However, the superior specificity of dCasNano vs. dSpCas9 observed in this study, particularly in the seed region, makes it an excellent candidate to fuse with other effector domains for precise genomic localization.

The durability of CasNano-mediated epigenetic editing differed between the two modalities tested. TET1-dCasNano-mediated demethylation and MECP2 reactivation were stably maintained for 5 months in 29-G hESCs, with persistent loss of CpG methylation confirmed by bisulfite sequencing (Figure S6). In contrast, DNMT3A-dCasNano-deposited methylation at the Snrpn promoter was not retained at 2 weeks in the GAPDH-Snrpn-EGFP HEK293T reporter (Figure S7); durable silencing was, therefore, not demonstrated for the DNMT3A module in this study. Several features of this reporter system may contribute to this outcome: it is a lentivirally integrated, multi-copy construct, in which the targeted Snrpn promoter sits adjacent to a constitutively active GAPDH promoter, and it is maintained in rapidly dividing HEK293T cells, in contrast to the single-copy, site-specific MECP2 knock-in of the 29-G hESC line. These interpretations remain to be tested, and establishing durable DNMT3A-dCasNano-mediated silencing will require evaluation in more appropriate models—for example, at single-copy endogenous loci, in non-dividing cellular contexts, or with sustained editor delivery such as AAV.

In conclusion, with the dual protein and sgRNA engineering approach, we transformed a minimally active natural protein into a powerful tool for genome engineering. CasNano's ultracompact size, high efficiency, robust specificity, and a therapeutically

advantageous PAM sequence makes it a valuable addition to the CRISPR toolbox. CasNano provides a new platform for programmable genome and epigenome engineering with potential application in cell and gene therapy and demonstrates a broadly applicable strategy to unlock the potential of other novel Cas effectors discovered through bioinformatic mining of natural Cas proteins.

MATERIALS AND METHODS

Bioinformatic mining of Cas12f homologs

To identify ultracompact Cas12f candidates for engineering, we employed a profile hidden Markov model (HMM)-based bioinformatic mining strategy. The sequence profile for the TnpB/Cas12f nuclease superfamily (Pfam ID: PF07282) was obtained from the InterPro database. This HMM profile was used to query the reference protein database of *Blautia lenta* (and related metagenomic datasets) through the hmmsearch tool from the HMMER suite. Significant hits were identified with a strict expectation value (E-value) cutoff of <0.01. To specifically isolate ultracompact effectors that maximize AAV packaging capacity, we applied a stringent length filtration step, retaining only candidate proteins with a size between 350 and 450 amino acids. This strategy efficiently excluded larger typical miniature Cas proteins (>500 aa), yielding a highly focused pool of ultracompact candidates. Among these, BlCas12f (~400 aa) was selected for further empirical screening and structural engineering on the basis of its distinct putative PAM-interacting domain sequences.

Plasmid construction and CasNano engineering

The codon-optimized sequence for the *Blautia lenta* Cas12f (BlCas12f) protein was synthesized as a gBlock gene fragment (Integrated DNA Technologies) (Table S2). This sequence was cloned into a mammalian expression vector under the control of a Ubiquitin C (UBC) promoter, which also encoded an N-terminal SV40 nuclear localization signal (NLS). Effector domains (VPR, DNMT3A, or TET1) were fused to the N or C terminus of dCasNano by using In-Fusion HD Cloning (Takara Bio) with a flexible linker. Protein engineering was performed via iterative site-directed and saturated mutagenesis. Specific point mutations were introduced by using PCR with primers containing the desired nucleotide changes, through Phusion Polymerase (Thermo Fisher).

The sgRNA expression vector was built on a vector with U6 promoter. The various sgRNA scaffold variants described in Figure 2 were synthesized as gBlocks (Integrated DNA Technologies) and cloned downstream of the U6 promoter (Table S3). The spacer sequences are listed in Table S4. Individual target-specific spacers (~20 nt) were incorporated by ligating annealed complementary oligonucleotides into the BsmBI-digested sgRNA expression vector. All final plasmid constructs were sequence-verified by nanopore sequencing.

Reporter cell line generation

The GAPDH-snrpn and Dazl-snrpn reporter HEK293T cell lines were a kind gift from Dr. Shawn Liu (Columbia University) and were previously described.[27] The TRE3G-RFP reporter line was generated by cloning a modified TRE3G promoter, containing seven TetO sequences flanked by a 5′ CCA PAM and a 3′ AGG PAM, into a lentiviral backbone. HEK293T cells were transduced with the resulting lentivirus, and a single clonal cell line exhibiting stable reporter expression was isolated for all subsequent experiments.

Cell culture and transfection

HEK293T cells and their derivative reporter lines were cultured in Dulbecco's modified Eagle's medium (DMEM, Gibco) supplemented with 10% fetal bovine serum (FBS, Gibco), 1× GlutaMAX (Gibco), 1× non-essential amino acids (NEAA, Gibco), and 1× sodium pyruvate (Gibco). The MECP2 dual-color reporter hESC line (29-G) was cultured in mTeSR Plus media (STEMCELL Technologies) on Geltrex (Gibco)-coated plates and passaged with ReLeSR (STEMCELL Technologies). All cells were maintained at 37°C in a 5% CO_2 incubator.

For HEK293T transfections, 50,000 cells were seeded per well in poly-L-lysine-coated 24-well plates one day prior to transfection. A total of 1.2 μg of plasmid DNA, containing Cas editor and sgRNA plasmids in a 3:1 mass ratio, was transfected through Lipofectamine 2000 (Invitrogen) according to the manufacturer's instructions. For hESCs, a reverse transfection was performed by plating 150,000 cells per well in mTeSR Plus with 1× CultureCEPT supplement (Gibco) on Geltrex-coated 24-well plates. A mix of 450 ng of Cas editor plasmid and 150 ng of sgRNA plasmid was delivered through 2 μL of Lipofectamine Stem (Invitrogen). Media was refreshed the following day. Cells were harvested for analysis 3 days post-transfection for transcriptional activation experiments or 6 days for epigenetic editing experiments. Equal-DNA-mass transfection was adopted in keeping with the convention of prior compact-Cas benchmarking studies (CasMINI vs. Cas12a[14] and CasX vs. SpCas9[13]); the underlying methodological considerations are discussed in detail in the discussion.

Flow cytometry

For flow cytometry analysis, transfected HEK293T and hESC lines were dissociated with trypsin (Gibco) or Accutase (Gibco), respectively. Cells were resuspended in Dulbecco's phosphate-buffered saline (Gibco) containing 2% FBS to generate single-cell suspensions. Samples were analyzed on a NovoCyte flow cytometer, and the resulting data were processed with the NovoExpress software.

Quantitative RT-PCR

Total RNA was extracted from transfected cells by using the RNeasy Plus Mini Kit (QIAGEN). cDNA was synthesized from the extracted RNA by using the SuperScript IV First-Strand Synthesis System (Invitrogen). Quantitative PCR (qPCR) was performed on a CFX96 Real-Time System (Bio-Rad) by using the Applied Biosystems SYBR Green Select Master Mix. Relative gene expression was calculated through the $\Delta\Delta$Cq method, with normalization to housekeeping gene beta-actin. Primers sequences for qPCR are listed in Table S5.

Bisulfite sequencing analysis

Genomic DNA was isolated from transfected cells with the DNeasy Blood & Tissue Kit (QIAGEN). The isolated DNA was then

subjected to bisulfite conversion with the EpiTect Fast DNA Bisulfite Kit (QIAGEN) according to the manufacturer's protocol. The targeted promoter region was amplified from the converted DNA by PCR, and the resulting amplicons were analyzed by Nanopore sequencing to determine the methylation level of individual CpG sites. Primers sequences for bisulfite sequencing are listed in Table S5.

Transcriptome sequencing

Total RNA was extracted from transfected cells with the RNeasy Plus Mini Kit (QIAGEN). Library preparation and sequencing were performed by Psomagen, Inc. Briefly, mRNA was enriched with poly(A) selection, and libraries were prepared for sequencing. Paired-end sequencing was performed on an Illumina NovaSeq platform. Raw reads were aligned to the human genome (hg38), and transcript abundance was quantified as transcripts per million (TPM).

Off-target site prediction

Candidate genome-wide off-target sites for the TRE3G-targeting spacer (5′-CTCCCTATCAGTGATAGAGA-3′) were identified with Cas-OFFinder v.2.4.1 against the human reference genome (GRCh38/hg38). Because dSpCas9 and dCasNano recognize PAMs on opposite sides of the protospacer, searches were performed separately for each system: 5′-CTCCCTATCAGTGATAGAGA-NGG-3′ for dSpCas9 and 5′-CCN-CTCCCTATCAGTGATAGAGA-3′ for dCasNano. Sites carrying up to four mismatches to the spacer were retained and mapped to annotated genes; intergenic sites were excluded, and the resulting gene sets were restricted to genes detected in the HEK293T RNA-seq dataset. Log2 fold-changes (targeting/scramble) at these genes were compared between the two editors with the Kolmogorov-Smirnov and Mann-Whitney tests.

Statistical analysis

All quantitative data are presented as mean ± standard deviation (SD) from a minimum of three biological replicates, unless otherwise specified. For endogenous gene activation experiments, data are represented as the geometric mean ± SD. Statistical significance for the comparison was determined using a two-tailed Student's *t* test. A p value of less than 0.05 was considered statistically significant.

DATA AND CODE AVAILABILITY

Plasmids encoding CasNano and the engineered sgRNA scaffolds will be made available through Addgene. Any other data supporting the findings of this study are available from the corresponding author upon reasonable request.

ACKNOWLEDGMENTS

This work is supported by NINDS SBIR 1R43NS139830.

AUTHOR CONTRIBUTIONS

S.L. conceived the idea for this project. Y.-T.T., S.L., and O.D. provided supervision. Y.-T.T. screened and identified the CasNano. Y.-T.T., D.K., and C.-W.H. designed, performed, and analyzed the experiments. Y.-T.T., D.K., and C.-W.H. wrote the manuscript. Y.-T.T. also performed computation and statistical analysis. S.L. and O.D. edited the manuscript. S.L., R.P., J.Q., and X.W. provided experimental materials. O.D., Y.-T.T., and S.L. participated in funding acquisition. The author order reflects the contribution level, from highest to lowest.

DECLARATION OF INTERESTS

D.K. and Y-T.T. are the inventors of CasNano. O.D. and S.L. have stock in Epitor Therapeutics.

DECLARATION OF GENERATIVE AI AND AI-ASSISTED TECHNOLOGIES IN THE MANUSCRIPT PREPARATION PROCESS

During the preparation of this work the author(s) used Gemini and Claude in order to check grammar and identify potential logical inconsistencies in the manuscript.

SUPPLEMENTAL INFORMATION

Supplemental information can be found online at https://doi.org/10.1016/j.omtn.2026.103106.

REFERENCES

1. McCutcheon, S.R., Rohm, D., Iglesias, N., and Gersbach, C.A. (2024). Epigenome editing technologies for discovery and medicine. Nat. Biotechnol. *42*, 1199–1217. https://doi.org/10.1038/s41587-024-02320-1.
2. Levesque, S., and Bauer, D.E. (2025). CRISPR-based therapeutic genome editing for inherited blood disorders. Nat. Rev. Drug Discov. *24*, 907–925.
3. Ma, Y., and Qi, L.S. (2024). The realization of CRISPR gene therapy. Nat. Chem. Biol. *20*, 791–795. https://doi.org/10.1038/s41589-024-01645-x.
4. Wang, D., Zhang, F., and Gao, G. (2020). CRISPR-Based Therapeutic Genome Editing: Strategies and In Vivo Delivery by AAV Vectors. Cell *181*, 136–150. https://doi.org/10.1016/j.cell.2020.03.023.
5. Doudna, J.A. (2020). The promise and challenge of therapeutic genome editing. Nature *578*, 229–236. https://doi.org/10.1038/s41586-020-1978-5.
6. Li, S., Hu, Y., Li, A., Lin, J., Hsieh, K., Schneiderman, Z., Zhang, P., Zhu, Y., Qiu, C., Kokkoli, E., et al. (2022). Payload distribution and capacity of mRNA lipid nanoparticles. Nat. Commun. *13*, 5561. https://doi.org/10.1038/s41467-022-33157-4.
7. Youssef, M., Hitti, C., Puppin Chaves Fulber, J., and Kamen, A.A. (2023). Enabling mRNA Therapeutics: Current Landscape and Challenges in Manufacturing. Biomolecules *13*, 1497. https://doi.org/10.3390/biom13101497.
8. Di, J., Du, Z., Wu, K., Jin, S., Wang, X., Li, T., and Xu, Y. (2022). Biodistribution and Non-linear Gene Expression of mRNA LNPs Affected by Delivery Route and Particle Size. Pharm. Res. *39*, 105–114. https://doi.org/10.1007/s11095-022-03166-5.
9. Pinheiro, R.G.R., Coutinho, A.J., Pinheiro, M., and Neves, A.R. (2021). Nanoparticles for Targeted Brain Drug Delivery: What Do We Know? Int. J. Mol. Sci. *22*, 11654. https://doi.org/10.3390/ijms222111654.
10. Wohlfart, S., Gelperina, S., and Kreuter, J. (2012). Transport of drugs across the blood-brain barrier by nanoparticles. J. Control. Release *161*, 264–273. https://doi.org/10.1016/j.jconrel.2011.08.017.
11. Jinek, M., Chylinski, K., Fonfara, I., Hauer, M., Doudna, J.A., and Charpentier, E. (2012). A programmable dual-RNA-guided DNA endonuclease in adaptive bacterial immunity. Science *337*, 816–821. https://doi.org/10.1126/science.1225829.
12. Zetsche, B., Gootenberg, J.S., Abudayyeh, O.O., Slaymaker, I.M., Makarova, K.S., Essletzbichler, P., Volz, S.E., Joung, J., van der Oost, J., Regev, A., et al. (2015). Cpf1 is a single RNA-guided endonuclease of a class 2 CRISPR-Cas system. Cell *163*, 759–771. https://doi.org/10.1016/j.cell.2015.09.038.
13. Liu, J.J., Orlova, N., Oakes, B.L., Ma, E., Spinner, H.B., Baney, K.L.M., Chuck, J., Tan, D., Knott, G.J., Harrington, L.B., et al. (2019). CasX enzymes comprise a distinct family of RNA-guided genome editors. Nature *566*, 218–223. https://doi.org/10.1038/s41586-019-0908-x.
14. Xu, X., Chemparathy, A., Zeng, L., Kempton, H.R., Shang, S., Nakamura, M., and Qi, L.S. (2021). Engineered miniature CRISPR-Cas system for mammalian genome regulation and editing. Mol. Cell *81*, 4333–4345.e4. https://doi.org/10.1016/j.molcel.2021.08.008.
15. Ran, F.A., Cong, L., Yan, W.X., Scott, D.A., Gootenberg, J.S., Kriz, A.J., Zetsche, B., Shalem, O., Wu, X., Makarova, K.S., et al. (2015). In vivo genome editing using Staphylococcus aureus Cas9. Nature *520*, 186–191. https://doi.org/10.1038/nature14299.

16. Hino, T., Omura, S.N., Nakagawa, R., Togashi, T., Takeda, S.N., Hiramoto, T., Tasaka, S., Hirano, H., Tokuyama, T., Uosaki, H., et al. (2023). An AsCas12f-based compact genome-editing tool derived by deep mutational scanning and structural analysis. Cell *186*, 4920–4935.e23. https://doi.org/10.1016/j.cell.2023.08.031.

17. Kong, X., Zhang, H., Li, G., Wang, Z., Kong, X., Wang, L., Xue, M., Zhang, W., Wang, Y., Lin, J., et al. (2023). Engineered CRISPR-OsCas12f1 and RhCas12f1 with robust activities and expanded target range for genome editing. Nat. Commun. *14*, 2046. https://doi.org/10.1038/s41467-023-37829-7.

18. Park, S.J., Ju, S., Jung, W.J., Jeong, T.Y., Yoon, D.E., Lee, J.H., Yang, J., Lee, H., Choi, J., Kim, H.S., et al. (2025). Robust genome editing activity and the applications of enhanced miniature CRISPR-Cas12f1. Nat. Commun. *16*, 677. https://doi.org/10.1038/s41467-025-56048-w.

19. Xiao, R., Li, Z., Wang, S., Han, R., and Chang, L. (2021). Structural basis for substrate recognition and cleavage by the dimerization-dependent CRISPR-Cas12f nuclease. Nucleic Acids Res. *49*, 4120–4128. https://doi.org/10.1093/nar/gkab179.

20. Wu, Z., Liu, D., Pan, D., Yu, H., Shi, J., Ma, J., Fu, W., Wang, Z., Zheng, Z., Qu, Y., et al. (2023). Structure and engineering of miniature Acidibacillus sulfuroxidans Cas12f1. Nat. Catal. *6*, 695–709. https://doi.org/10.1038/s41929-023-00995-4.

21. Wu, T., Liu, C., Zou, S., Lyu, R., Yang, B., Yan, H., Zhao, M., and Tang, W. (2024). Author Correction: An engineered hypercompact CRISPR-Cas12f system with boosted gene-editing activity. Nat. Chem. Biol. *20*, 129. https://doi.org/10.1038/s41589-023-01508-x.

22. Totaro, M.G., Vide, U., Zausinger, R., Winkler, A., and Oberdorfer, G. (2024). ESM-scan-A tool to guide amino acid substitutions. Protein Sci. *33*, e5221. https://doi.org/10.1002/pro.5221.

23. Chen, P., Zhou, J., Liu, H., Zhou, E., He, B., Wu, Y., Wang, H., Sun, Z., Paek, C., Lei, J., et al. (2024). Engineering of Cas12a nuclease variants with enhanced genome-editing specificity. PLoS Biol. *22*, e3002514. https://doi.org/10.1371/journal.pbio.3002514.

24. Gao, L., Cox, D.B.T., Yan, W.X., Manteiga, J.C., Schneider, M.W., Yamano, T., Nishimasu, H., Nureki, O., Crosetto, N., and Zhang, F. (2017). Engineered Cpf1 variants with altered PAM specificities. Nat. Biotechnol. *35*, 789–792. https://doi.org/10.1038/nbt.3900.

25. Huang, H., Huang, G., Tan, Z., Hu, Y., Shan, L., Zhou, J., Zhang, X., Ma, S., Lv, W., Huang, T., et al. (2022). Engineered Cas12a-Plus nuclease enables gene editing with enhanced activity and specificity. BMC Biol. *20*, 91. https://doi.org/10.1186/s12915-022-01296-1.

26. Zhang, L., Zuris, J.A., Viswanathan, R., Edelstein, J.N., Turk, R., Thommandru, B., Rube, H.T., Glenn, S.E., Collingwood, M.A., Bode, N.M., et al. (2021). Author Correction: AsCas12a ultra nuclease facilitates the rapid generation of therapeutic cell medicines. Nat. Commun. *12*, 4500. https://doi.org/10.1038/s41467-021-24770-w.

27. Liu, X.S., Wu, H., Ji, X., Stelzer, Y., Wu, X., Czauderna, S., Shu, J., Dadon, D., Young, R.A., and Jaenisch, R. (2016). Editing DNA Methylation in the Mammalian Genome. Cell *167*, 233–247.e17. https://doi.org/10.1016/j.cell.2016.08.056.

28. Binz, R.L., Tian, E., Sadhukhan, R., Zhou, D., Hauer-Jensen, M., and Pathak, R. (2019). Identification of novel breakpoints for locus- and region-specific translocations in 293 cells by molecular cytogenetics before and after irradiation. Sci. Rep. *9*, 10554. https://doi.org/10.1038/s41598-019-47002-0.

29. Qian, J., Guan, X., Xie, B., Xu, C., Niu, J., Tang, X., Li, C.H., Colecraft, H.M., Jaenisch, R., and Liu, X.S. (2023). Multiplex epigenome editing of MECP2 to rescue Rett syndrome neurons. Sci. Transl. Med. *15*, eadd4666. https://doi.org/10.1126/scitranslmed.add4666.

30. Wu, T., Liu, C., Zou, S., Lyu, R., Yang, B., Yan, H., Zhao, M., and Tang, W. (2023). An engineered hypercompact CRISPR-Cas12f system with boosted gene-editing activity. Nat. Chem. Biol. *19*, 1384–1393. https://doi.org/10.1038/s41589-023-01380-9.

31. Xin, C., Yin, J., Yuan, S., Ou, L., Liu, M., Zhang, W., and Hu, J. (2022). Comprehensive assessment of miniature CRISPR-Cas12f nucleases for gene disruption. Nat. Commun. *13*, 5623. https://doi.org/10.1038/s41467-022-33346-1.

OMTN, Volume *37*

Supplemental information

CasNano: An ultracompact CRISPR-Cas12f with a GC-rich PAM for epigenome editing with therapeutic potential

Dongkyeong Kim, Chia-Wei Huang, Richard Pan, Junming Qian, Xuebing Wu, Orrin Devinsky, Yi-Ting Tsai, and Shawn Liu

Table S1. The 22 candidate residues evaluated in the initial activity screen. The 10 residues with >80% wild-type activity (advanced to iterative engineering) are underlined.

Order	Amino acid
1	Y48
2	A50
3	T66
4	S98
5	T99
6	E109
7	N114
8	F119
9	S131
10	G176
11	E181
12	D183
13	K184
14	N195
15	K220
16	A223
17	G266
18	A276
19	Y306
20	T307
21	K318
22	D353

Table S2. CasNano Sequences

**Name**	**Description**	**Protein sequence**	**Lengt h**
WT BlCas12f	The original, unmodified wild-type Cas12f protein sequence derived from *Blautia lenta*.	MITVRKVKLIVNSEEAEEINRTYKFIRD<br>SMYAQYQGLNRCMGYLLSGYYANG<br>MDIKSDGFKNHMKTIKNSLNIFDDINF<br>GIGIDSKSAITQKVKKDFSTSLKNGLA<br>KGERGATNYKRNFPLMTRGRDVKIS<br>YLEDTNTFVIKWVNKIEFKVILGQKDNI<br>ELSHTLHKIINKEYTLGQCTFEFDKNN<br>KLLLALNINIPDNLISKNKEIIPGRVLGV<br>DLGVKVPAMICLNDNTFIKKSIGSYNE<br>FFKVRSQFKARRERLYKQLESSNGG<br>KGRKHKLKATMQFRDKEKNFARTYN<br>HFLSKNIIEFAQKYTCETINLEELNKKG<br>FDNNLLGKWGYYQLQSMIEYKAERV<br>GIKVKYVDPAFTSQTCSKCGYVDEEN<br>RITQDKFECQKCGFTLNADHNAAINIA<br>RK	400
CasNano V11	The final engineered CasNano variant incorporating all optimized mutations for enhanced targeting efficiency.	MITVRKVKLRVNSEEAEEINRTYKFIR<br>DSMYAQYQGLNRCMGYLLSGYYANG<br>MDIKSDGFKNHMKTIKNSLNIFDKINF<br>GIGIDSKSAITQKVKKDFSTSLKNGLA<br>KGERNATNYKRNFPLMTRGRDVKIC<br>YLEDTNTFVIKWVNKIEFKVILGQKDN<br>RELSHTLHKIINKEYTLGQCTFQFTKN<br>NKLLLALNIKVPDNLISKNKEIIPGRVL<br>GVDLGVKVPAMICLNDNTFIKKSIGSY<br>NEFFKVRSQFKARRERLYKQLESSN<br>GGKGRKHKLKATMQFRDKEKNFART<br>YNHFLSKNIIEFAQKKKCETINLEELNK<br>KGFDNNLLGKWGYYQLQSMIEYKAE<br>RVGIKVKYVDPAFTSQTCSKCGYVDE<br>ENRITQDKFECQKCGFTLNADHNAAI<br>NIARK	400

dCasNano V11	The catalytically dead version of CasNano V11 (harboring the D216A and D390A mutations). This variant lacks nuclease cleavage activity but retains full DNA-binding capability, serving as the versatile platform for fusion with transcriptional and epigenetic effectors (e.g., VPR, DNMT3A, TET1).	MITVRKVKLRVNSEEAEEINRTYKFIR<br>DSMYAQYQGLNRCMGYLLSGYYANG<br>MDIKSDGFKNHMKTIKNSLNIFDKINF<br>GIGIDSKSAITQKVKKDFSTSLKNGLA<br>KGERNATNYKRNFPLMTRGRDVKIC<br>YLEDTNTFVIKWVNKIEFKVILGQKDN<br>RELSHTLHKIINKEYTLGQCTFQFTKN<br>NKLLLALNIKVPDNLISKNKEIIPGRVL<br>GVALGVKVPAMICLNDNTFIKKSIGSY<br>NEFFKVRSQFKARRERLYKQLESSN<br>GGKGRKHKLKATMQFRDKEKNFART<br>YNHFLSKNIIEFAQKKKCETINLEELNK<br>KGFDNNLLGKWGYYQLQSMIEYKAE<br>RVGIKVKYVDPAFTSQTCSKCGYVDE<br>ENRITQDKFECQKCGFTLNAAHNAAI<br>NIARK	400

Table S3. gRNA Scaffold

**Variant**	**Scaffold sequence**	**Length**
WT full length	CAGGATAAATTTGAATGCCAAAAGTGTGGCTTTACTCTTAA TGCAGATCATAATGCAGCAATAAATATTGCACGTAAGTAAA CATTGATAAAACTAACCTTTATTTACAAATTTAAAATAATGC AATATAATTGTATTGATGTTATATATAAATATATAGCAGTTA CGGTAACTTAAAGTGCCGAAGGCTGAGGAGATGGATTAAA TATATAAGGTTTTGACCAACTATATACCATCATCACTCGGT AAGGGTTAATCCTAACAATGTGTGACCGTTAGGCGTTCCA AAATTGAATAATTATATTAGTTTATTGTCTATCATAGATAGT GTAAAAAACACAGGGGGTAGACAAAATAAGTAATATAAGT ATATGGTAGCTA	381
Variant 1	TAATGCAATATAATTGTATTGATGTTATATATAAATATATAG CAGTTACGGTAACTTAAAGTGCCGAAGGCTGAGGAGATG GATTAAATATATAAGGTTTTGACCAACTATATACCATCATCA CTCGGTAAGGGTTAATCCTAACAATGTGTGACCGTTAGGC GTTCCAAAGAAATGGAATGTTAAT	187
Variant 3	GACGGTAACTTAAAGTGCCGAAGGCTGAGGAGATGGATTA AATATATAAGGGTTTGCCCAACTATATACCATCATCACTCG GTAAGGGTTAATCCTAACAATGTGTGACCGTTAGGCGTTC CAAAGAAATGGAACGTTAAT	141
Variant 5	GACGGTAACTTAAAGTGCCGAAGGCTGAGGAGATGGATTA AATATATAAGGGTTTGCCCAACTATATACCATCATCACTCG GTAAGGGTTAATCCTAACAATGTGTGACCGTTAGGCGTTC CAAAGAAATGGAATGTTAAT	141
Variant 14	GACGGTAACTTAAAGTGCCGAAGGCTGAGGAGATGGATTA AATATATAAGGTTTTGACCAACTATATACCATCATCACTCG GTAAGGGTTAATCCTAACAATGTGTGACCGTTAGGCGTTC CAAAGAAATGGAACGTTAAT	141
Variant 18	GCGTAACTTAAAGTGCGAAGGCTGAGGAGATGGATTAAAT ATATAAGGTTTCGACCAACTATATACCATCATCACTCGGTA AGGGTTAATCCTAACAATGTGTGACCGTTAGGCGTTCCGA AAGGAACGTTAAT	134

Table S4. gRNAs

	Target	gRNA spacer sequence	Lenth
1	Scramble	GATATGCTTCTAGATGAGAA	20
2	TetO(sense)	CTCCCTATCAGTGATAGAGA	20
3	TetO(antisense)	TCTCTATCACTGATAGGGAG	20
4	HBG1(sense)	GGCTAAACTCCACCCATGGGT	21
5	HBG1(sense)	ATAGTCTTAGAGTATCCAGTG	21
6	HBG1(sense)	GTGAGGCCAGGGGCCGGCGGC	21
7	HBG1(sense)	TGGGTCATTTCACAGAGG	18
8	HBG1(antisense)	ACCCATGGGTGGAGTTTAGCC	21
9	HBG1(antisense)	CACTGGATACTCTAAGACTAT	21
10	HBG1(antisense)	GCCGCCGGCCCCTGGCCTCAC	21
11	HBG1(antisense)	CCTCTGTGAAATGACCCA	18
12	IL1RN(sense)	TGTACTCTCTGAGGTGCTC	19
13	IL1RN(sense)	CCTGGAAACTGGGCCTGCT	19
14	IL1RN(sense)	GAGTCACCCTCCTGGAAACT	20
15	IL1RN(sense)	CTGCCTTGCTGCAGTCACAGAA	22
16	IL1RN(antisense)	GAGCACCTCAGAGAGTACA	19
17	IL1RN(antisense)	AGCAGGCCCAGTTTCCAGG	19
18	IL1RN(antisense)	AGTTTCCAGGAGGGTGACTC	20
19	IL1RN(antisense)	TTCTGTGACTGCAGCAAGGCAG	22
20	GAPDH-mSnrpn	TTGCCTGGGACGCATGCG	18
21	GAPDH-mSnrpn	TTGCGGCAAGACTAGCGC	18
22	GAPDH-mSnrpn	GCTGACCTTCCTCGCTCC	18
23	DAZL-Snrpn	TTGCCTGGGACGCATGCG	18
24	DAZL-Snrpn	TTGCGGCAAGACTAGCGC	18
25	DAZL-Snrpn	GCTGACCTTCCTCGCTCC	18
26	MeCP2	CAGCCCTCTCTCCGAGAGG	19
27	MeCP2	CTCACAGTCTCTCCTCCTCG	20
28	MeCP2	CTAAACCAGTCCCTCCGCGC	20

Table S5. Primers

Primers	Sequence	Length
IL1RN-F	GGAATCCATGGAGGGAAGAT	20
IL1RN-R	TGTTCTCGCTCAGGTCAGTG	20
HBG1-F	GTCCTGGTCACCAGAGCCT	19
HBG1-R	ACCACTGCTAACTGAAAGAGAC	22
GAPDH-Snrpn-R	TTAGGTTAGTTGAGAGAATTTTAGAATA	28
GAPDH-Snrpn-F	CTCCTTAATAATAACCATATTATCCTCC	28
DAZL-Snrpn-R	GGGAGGAGTAGAGAGTATTAGGG	23
DAZL-Snrpn-F	CTCCTCGCCCTTACTCACCATAATAAC	27

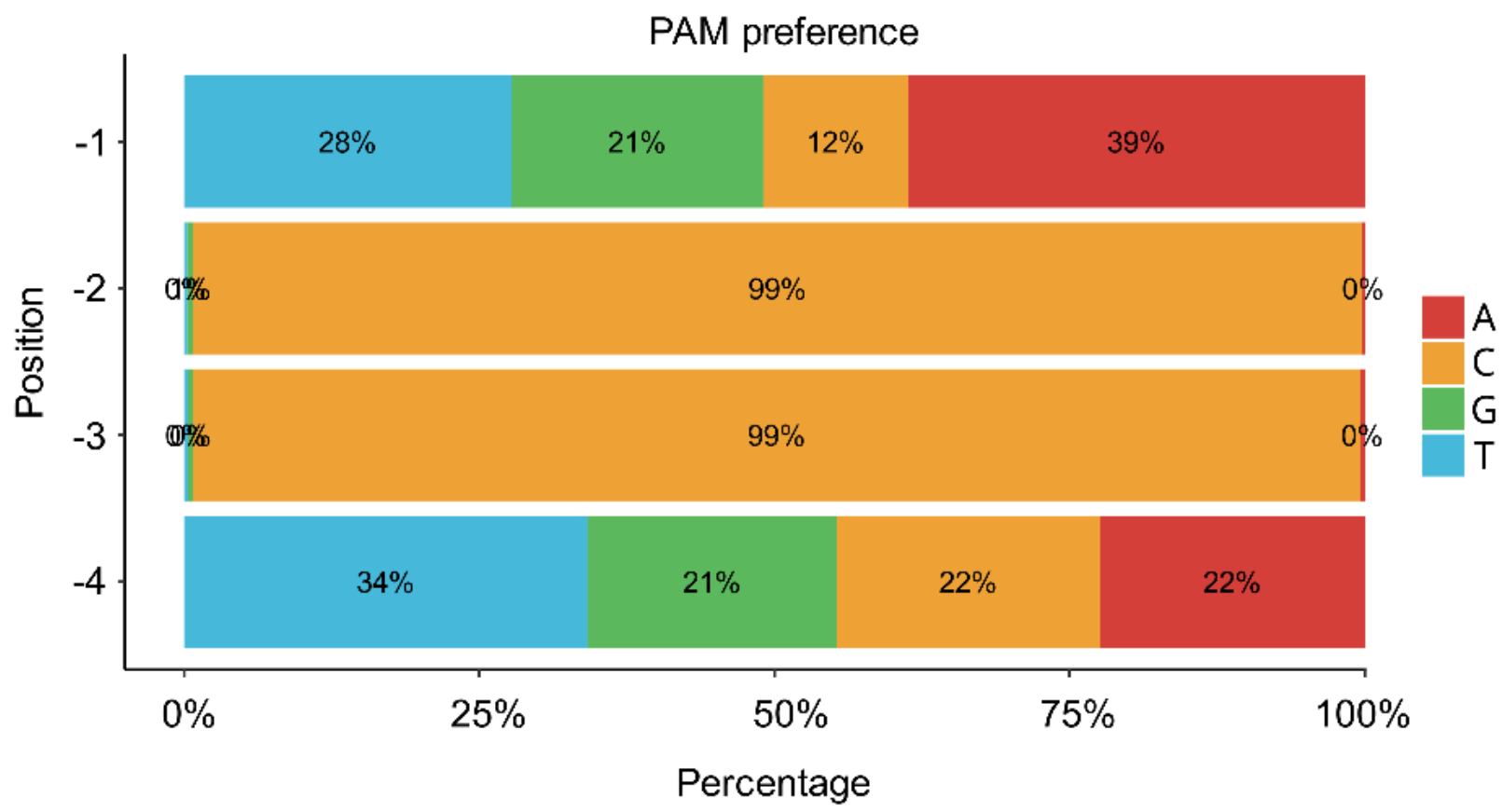

Figure S1. Characterization of CasNano PAM Preference.

Bar chart showing the nucleotide preference at the -1 to -4 positions of the protospacer adjacent motif (PAM). A library of reporter plasmids, each containing a unique 4-bp 5'-NNNN-3' sequence upstream of the TetO target site, was co-transfected into HEK293T cells with dCasNano-VPR and a TetO-targeting sgRNA. RFP intensity was measured by flow cytometry, and the relative activity for each nucleotide at each position was calculated to determine the preference. The results show a strict requirement for Cytosine (C) at positions -2 and -3.

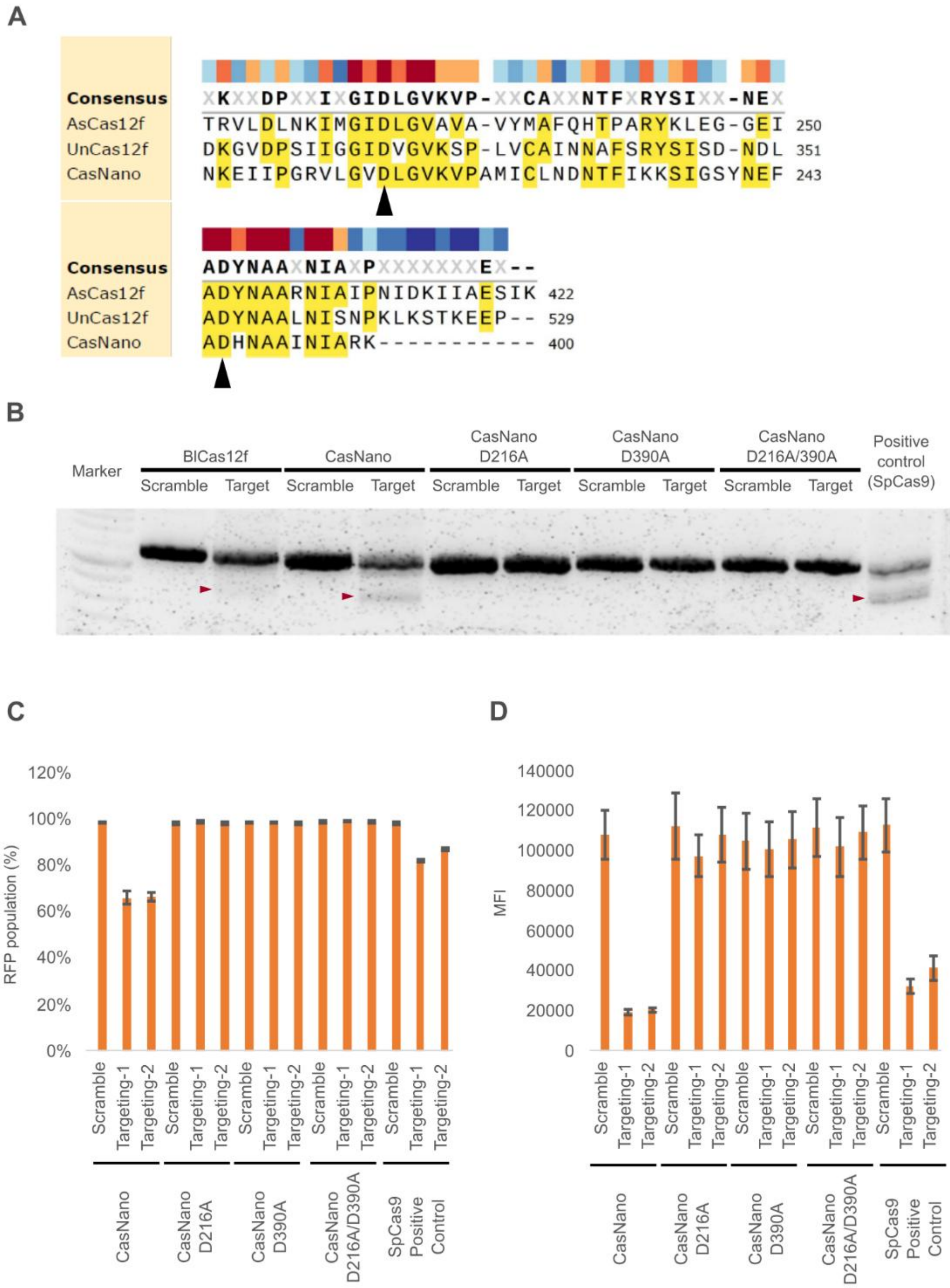
A
Consensus XKXXDPXXIXGIDLGVKVP-XXCAXXNTFXRYSIXX-NEX
AsCas12f TRVLDLNKIMGIDLGVAVA-VYMAFQHTPARYKLEG-GEI 250
UnCas12f DKGVDPSIIGGIDVGVKSP-LVCAINNAFSRYSISD-NDL 351
CasNano NKEIIPGRVLGVDLGVKVPAMICLNDNTFIKKSIGSYNEF 243
Consensus ADYNAAXNIAXPXXXXXXXEX--
AsCas12f ADYNAARNIAIPNIDKIIAESIK 422
UnCas12f ADYNAALNISNPKLKSTKEEP-- 529
CasNano ADHNAAINIARK----------- 400
B
Marker
BlCas12f
CasNano
CasNano D216A
CasNano D390A
CasNano D216A/390A
Positive control (SpCas9)
Scramble
Target
C
RFP population (%)
120%
100%
80%
60%
40%
20%
0%
Scramble
Targeting-1
Targeting-2
CasNano
CasNano D216A
CasNano D390A
CasNano D216A/D390A
SpCas9 Positive Control
D
MFI
140000
120000
100000
80000
60000
40000
20000
0
Scramble
Targeting-1
Targeting-2
CasNano
CasNano D216A
CasNano D390A
CasNano D216A/D390A
SpCas9 Positive Control

Figure S2. Determination of Catalytic Amino Acids of CasNano Nuclease Activity.

(A) Protein sequence alignment of the RuvC domain from CasNano with AsCas12f and UnCas12f, highlighting the predicted catalytic aspartate residues (D216 and D390, indicated).

(B) Surveyor nuclease assay showing indel formation. Wild-type BlCas12f and the engineered CasNano (V11) were transfected into HEK293 cells with either a scramble control sgRNA or two different sgRNAs targeting the RFP gene. Cleaved DNA fragments indicative of NHEJ are marked with an arrow. Semi-quantitative densitometric analysis using ImageJ confirmed that the indel frequency of wild-type BlCas12f was near background levels (~0.10%). The engineered CasNano variant exhibited a weak nuclease activity (~7.45%), which was completely abolished (0%) by the D216A and/or D390A mutations. SpCas9 was used as a positive control (~28.90%).

(C and D) Quantification of nuclease activity via a functional knockout assay in TetO-RFP reporter cells. Bar charts show the percentage of RFP-positive cells (C) and the mean fluorescence intensity (MFI) of RFP (D) after transfection with nuclease-active CasNano or variants with single (D216A, D390A) or double (D216A/D390A) catalytic mutations. A decrease in RFP signal indicates successful cleavage and NHEJ-mediated gene disruption. Nuclease-active SpCas9 serves as a positive control.

All data are represented as mean ± SD for n = 3 biological replicates.

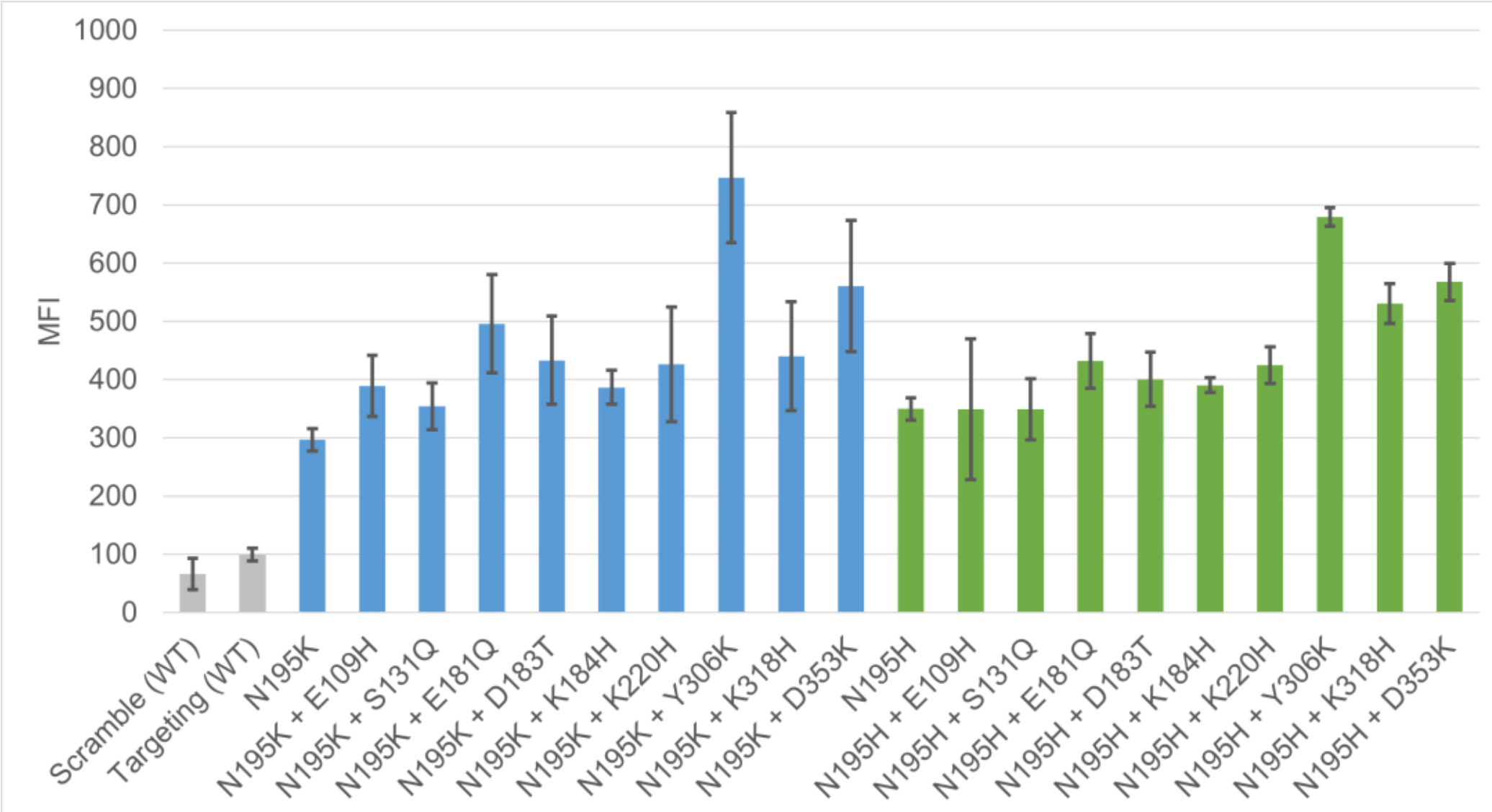

Figure S3. Combinatorial pairwise testing of N195K vs N195H as the V1 scaffold of dBlCas12f-VPR.

Reporter activation of the indicated dBlCas12f-VPR variants on the TRE3G-RFP reporter in HEK293T cells, measured by flow cytometry 72 h post-transfection. Bars show the mean fluorescence intensity (MFI) of RFP. Gray: wild-type dBlCas12f-VPR with either a scramble or a targeting sgRNA, providing the scramble background and the unengineered baseline-activity controls. Blue: the N195K-base series, comprising the single mutation N195K and N195K paired in turn with each of the nine top single-mutation candidates retained from saturated mutagenesis (E109H, S131Q, E181Q, D183T, K184H, K220H, Y306K, K318H, D353K; Figure 1B). Green: the corresponding N195H-base series, with the same nine pairings on the alternative N195H base. Data are mean ± SD for n = 2 biological replicates.

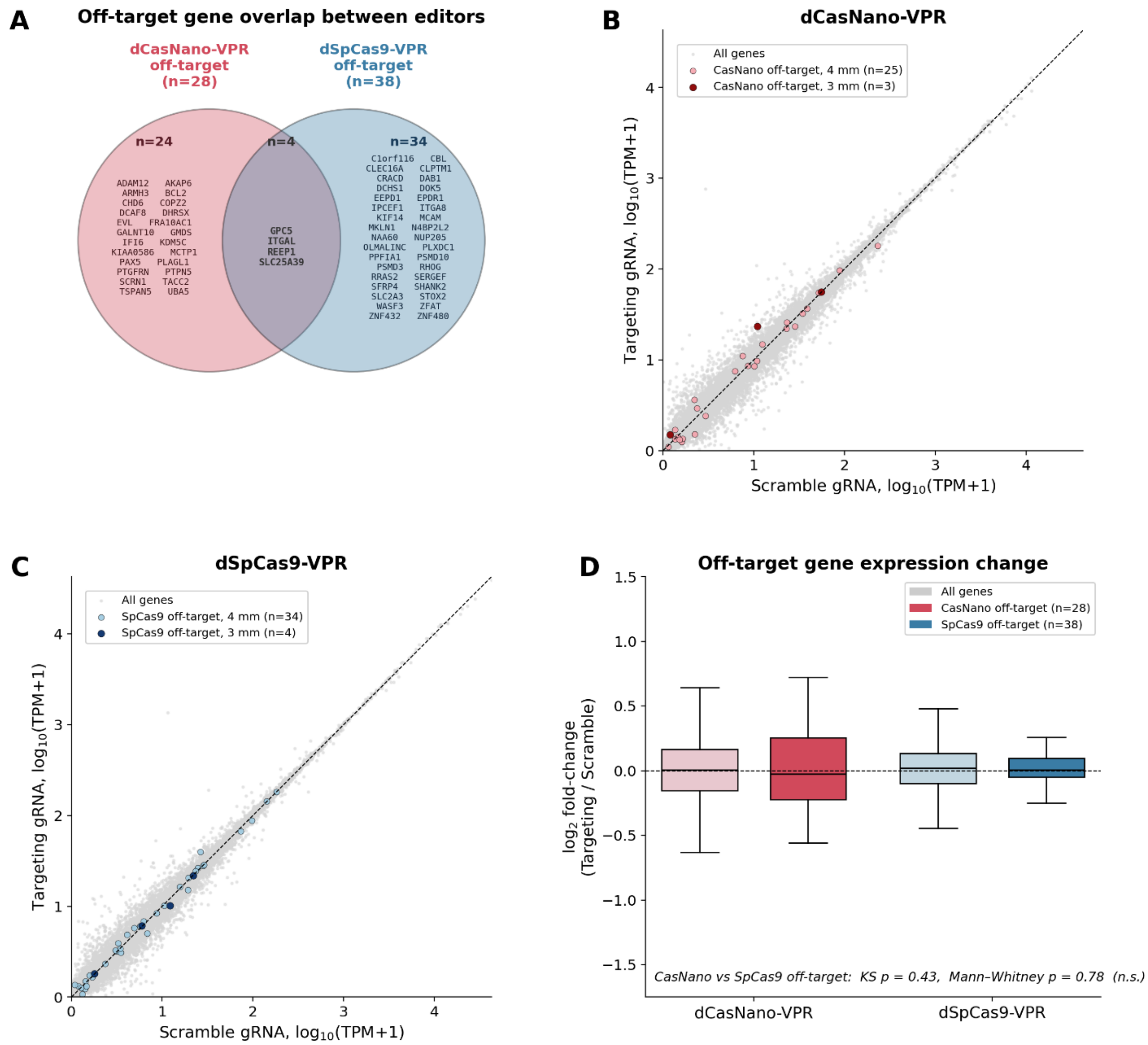

Figure S4. Genome-wide transcriptional specificity of dCasNano-VPR and dSpCas9-VPR at predicted off-target loci.

(A) Overlap between the predicted off-target gene sets of the two editors. Candidate sites within four mismatches of the shared spacer were identified separately for each system, reflecting their distinct PAM requirements. (B and C) Gene expression (log10(TPM+1)) under Targeting versus Scramble gRNA conditions for dCasNano-VPR (B) and dSpCas9-VPR (C). Each editor's own predicted off-target genes are highlighted by mismatch number; the remaining transcriptome is shown in gray. (D) Box plots of log2 fold-change (Targeting/Scramble) for all genes and for each editor's predicted off-target genes. Kolmogorov–Smirnov and Mann–Whitney p values compare the two off-target gene sets.

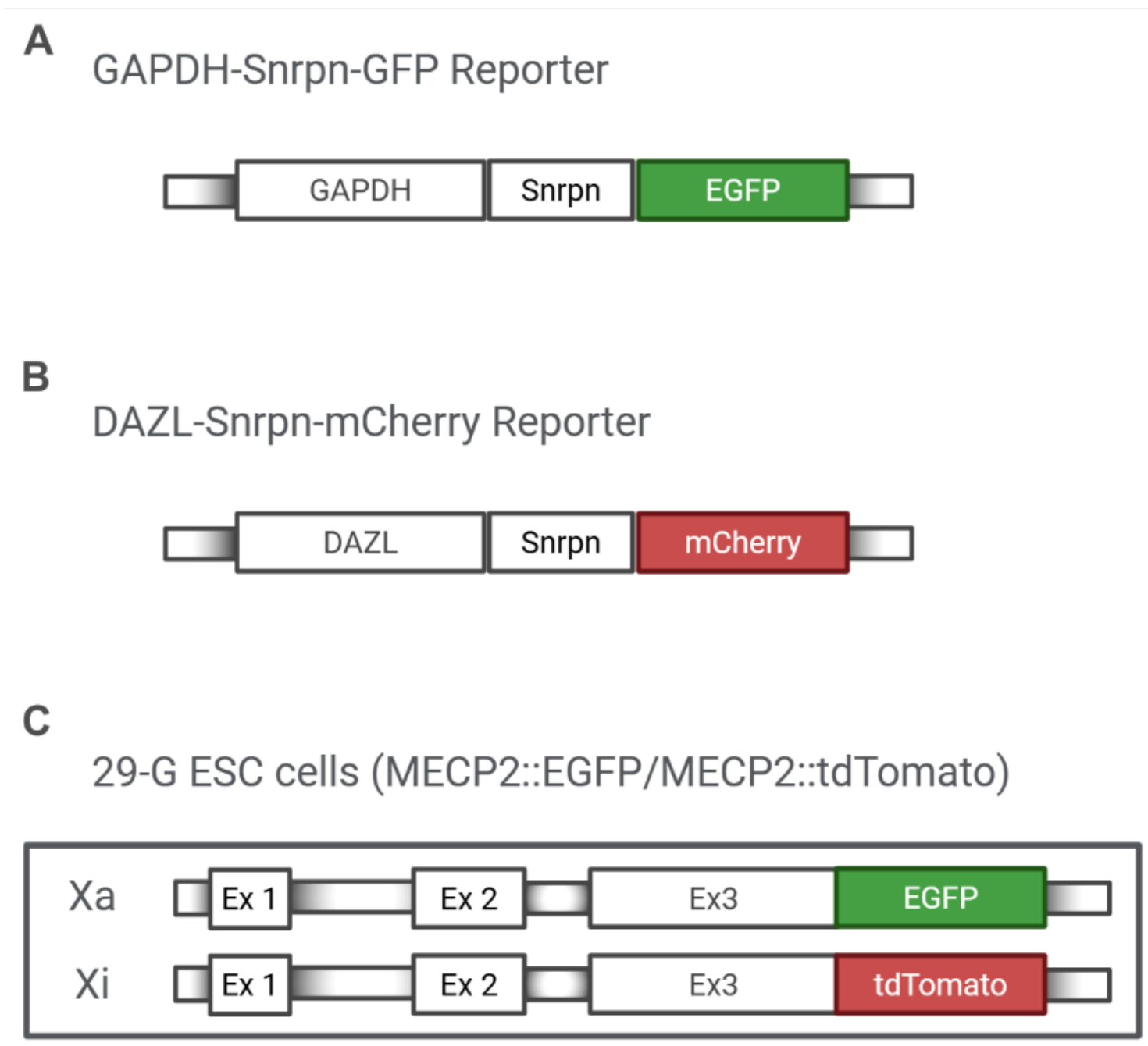

Figure S5. Schematics of Reporter Constructs Used for Epigenetic Editing.

(A) Diagram of the GAPDH-Snrpn-EGFP reporter construct integrated into the HEK293 genome. The constitutively active GAPDH promoter keeps the adjacent Snrpn promoter from silencing and maintain the expression of a downstream EGFP reporter.

(B) Diagram of the DAZL-Snrpn-mCherry reporter construct. The heavily methylated DAZL promoter leads to silencing of the linked Snrpn promoter and the mCherry reporter.

(C) Diagram of the 29-G hESC reporter line for the *MECP2* locus. An EGFP reporter is fused in-frame to the end of exon 3 on the active X chromosome (Xa), while a tdTomato reporter is fused to the end of exon 3 on the inactive X chromosome (Xi), which is silenced by DNA methylation.

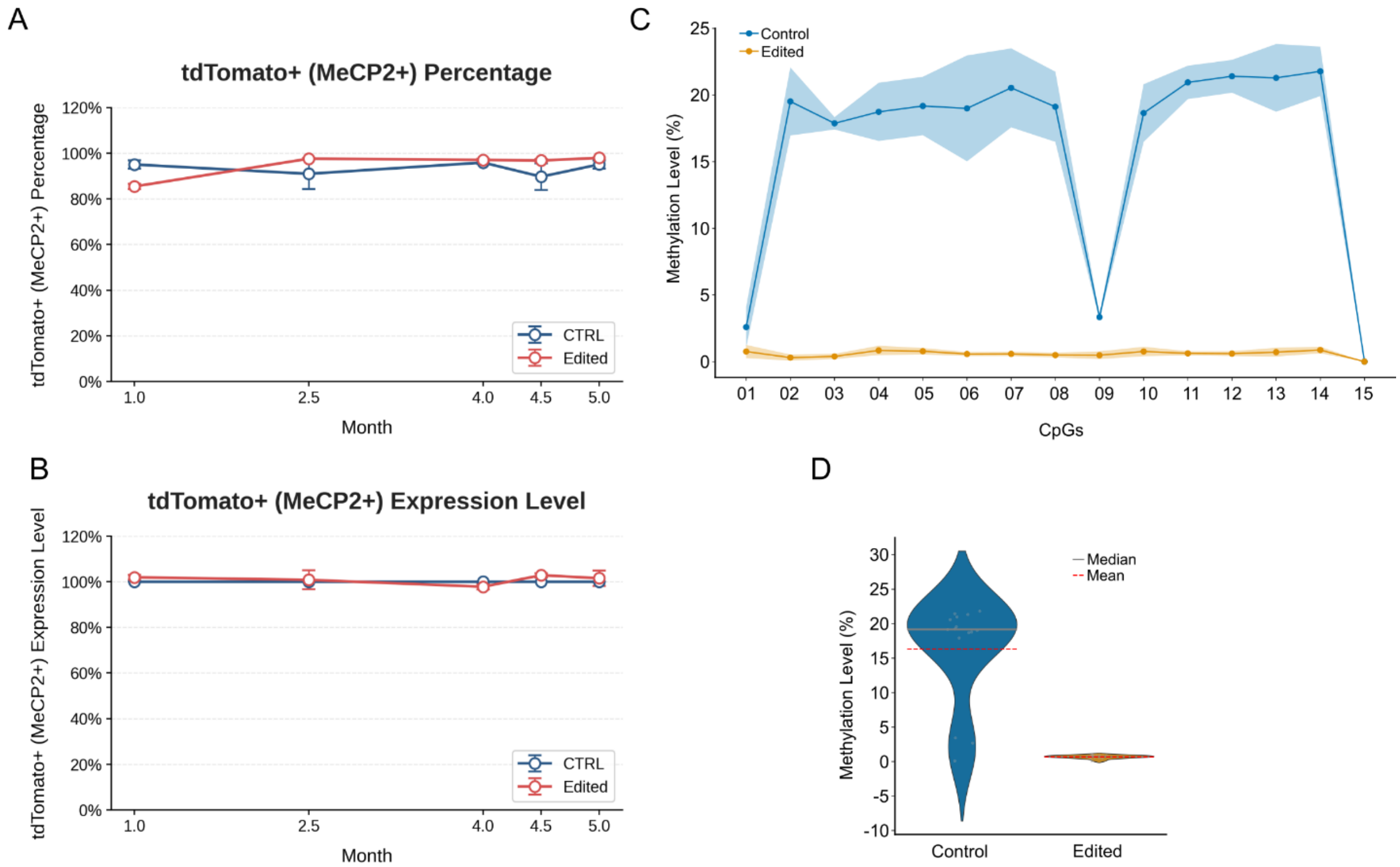

Figure S6. Long-term durability of CasNano-mediated targeted demethylation.

(A and B) Time-course flow cytometry analysis of 29-G hESCs edited with TET1-dCasNano, monitored over a 5-month period. (A) Percentage of tdTomato (MECP2)-positive cells in the culture. (B) Relative tdTomato (MECP2) expression level (MFI). The control for (A) and (B) is the 29-R hESC line, in which the MECP2-tdTomato allele is constitutively expressed; it defines full reactivation, and values in (B) are expressed relative to this line (29-R hESC). (C and D) Bisulfite sequencing analysis of MECP2 promoter CpG methylation at the 5-month time point in TET1-dCasNano-edited versus unedited 29-G hESCs. The control for (C) and (D) is unedited 29-G hESCs, which retain a methylated MECP2 promoter and a silenced tdTomato allele. (C) Per-CpG methylation level (%) across 15 anchor CpG positions spanning the targeted MECP2 promoter region; lines indicate group means, shaded ribbons indicate SD across biological replicates. (D)

Violin plot of methylation level (%) across all profiled CpG positions, with median and mean indicated. (N=5)

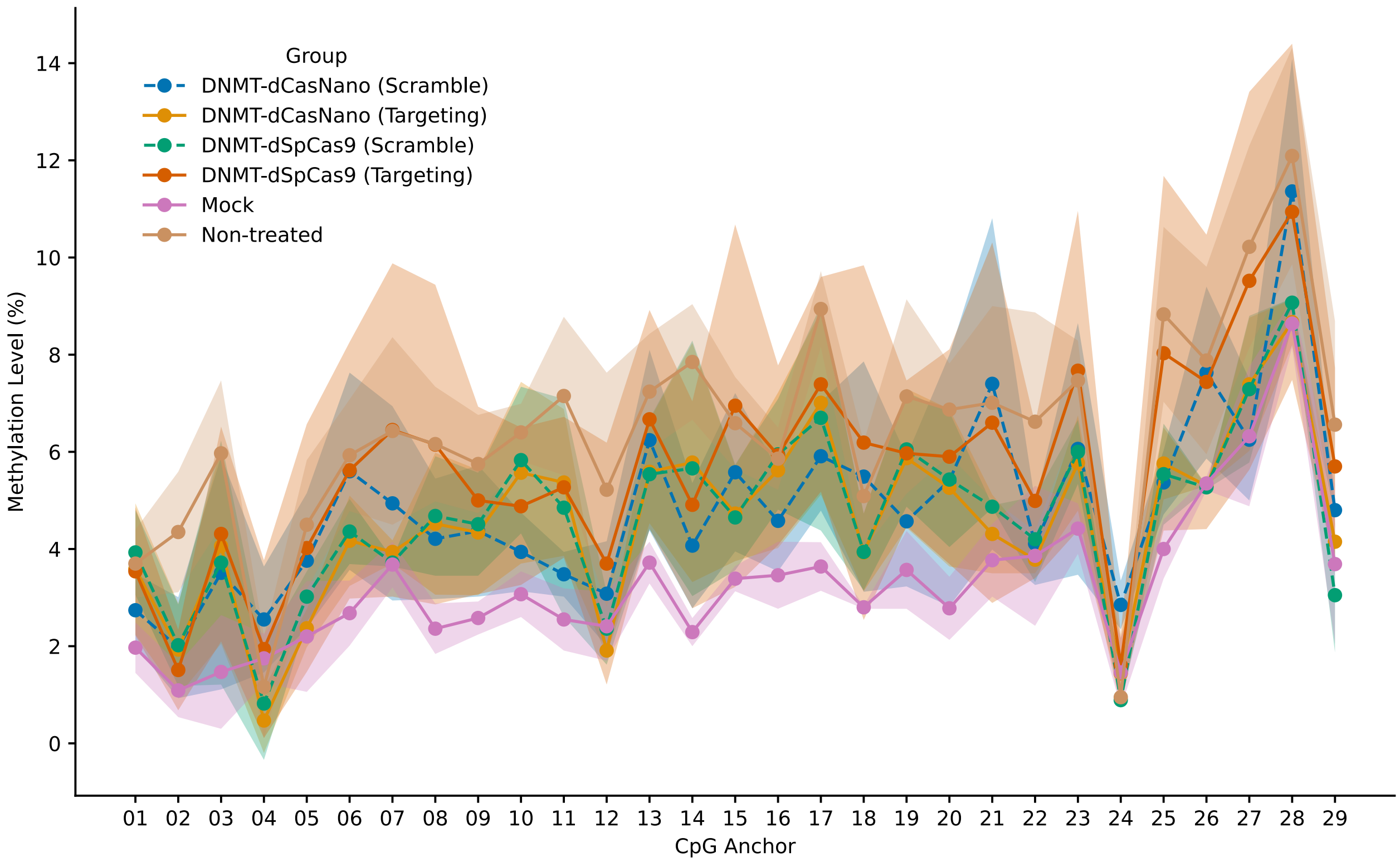

Figure S7. Durability assessment of DNMT3A-dCasNano-mediated methylation in HEK293T cells.

Methylation level (%) at 29 anchor CpG positions across the Snrpn promoter region in the GAPDH-Snrpn-EGFP HEK293T reporter line at 2 weeks post-transient transfection. Lines indicate group means; shaded ribbons indicate SD across biological replicates.
